# Relationships between House Characteristics and Exposures to Metal(loid)s and Synthetic Organic Contaminants Evaluated Using Settled Indoor Dust

**DOI:** 10.3390/ijerph191610329

**Published:** 2022-08-19

**Authors:** Pat E. Rasmussen, Cariton Kubwabo, H. David Gardner, Christine Levesque, Suzanne Beauchemin

**Affiliations:** 1 Environmental Health Science and Research Bureau, HECS Branch, Health Canada, Ottawa, ON K1A 0K9, Canada; 2 Department of Earth and Environmental Sciences, University of Ottawa, Ottawa, ON K1N 9A7, Canada

**Keywords:** building characteristics, indoor chemistry, residential sources, consumer products, chemical loading

## Abstract

This study investigates associations between house characteristics and chemical contaminants in house dust, collected under the nationally representative Canadian House Dust Study (2007–2010). Vacuum samples (<80 µm fraction) were analysed for over 200 synthetic organic compounds and metal(loid)s. Spearman rank correlations between contaminant concentrations in dust and presence of children and pets, types of flooring, heating styles and other characteristics suggested a number of indoor sources, pointing to future research directions. Numerous synthetic organics were significantly associated with reported use of room deodorizers and with the presence of cats in the home. Hardwood flooring, which is a manufactured wood product, emerged as a source of metal(loid)s, phthalates, organophosphate flame retardants/plasticizers, and obsolete organochlorine pesticides such as ∑DDT (but not halogenated flame retardants). Many metal(loid)s were significantly correlated with flame-retardant compounds used in building materials and heating systems. Components of heating appliances and heat distribution systems appeared to contribute heat-resistant chemicals and alloys to settled dust. Carpets displayed a dual role as both a source and repository of dust-borne contaminants. Contaminant loadings (<80 µm fraction) were significantly elevated in heavily carpeted homes, particularly those located near industry. Depending on the chemical (and its source), the results show that increased dust mass loading may enrich or dilute chemical concentrations in dust. Research is needed to improve the characterisation of hidden indoor sources such as flame retardants used in building materials and heating systems, or undisclosed ingredients used in common household products, such as air fresheners and products used for companion animals.

## 1. Introduction

Settled house dust is a useful environmental medium for evaluating indoor concentrations of chemical contaminants for exposure assessment and for identifying and mitigating contaminant sources [1,2]. Dust monitoring is most commonly used for estimating ingestion exposures, particularly for children who frequently put their hands and other dust-contaminated objects in their mouths [3,4], but is also used for estimating dermal and inhalation exposures [5,6,7]. Haines et al. [8] concluded that measuring dust from carpets and floors can be a better surrogate for long-term exposure than short-term air samples. Mechanisms for the transfer of contaminants from indoor sources to settled dust include volatilisation and subsequent sorption to dust, and degradation of products and coatings followed by deposition of the abraded particles [9,10,11]. Indoor sources include building materials such as vinyl flooring, synthetic carpets and composite wood; consumer products such as electronics, personal care products, foam cushions, plastics, toys, scented cleaning agents and air fresheners [12,13,14]; and emissions from smoking, cooking and other combustion sources, including wood-burning, candles and flint lighters [2,15,16,17]. Studies have demonstrated direct transfer of chemicals from source product to dust; for example, Bi et al. [18] found that the concentration of benzyl butyl phthalate (BBzP) in dust in direct contact with vinyl flooring was 10-fold higher than dust from non-vinyl surfaces in the same home.

A complex combination of factors influences contaminant concentrations in settled dust, evidenced by order-of-magnitude variations within rooms and between rooms in the same house [10,19,20]. The influence of indoor spatial heterogeneity can be minimized by collecting composite dust samples or averaging dust results from a minimum of four rooms per home [20,21]. The selection of dust sampling methodology, however, depends on the purpose of the research, available resources, and other practical considerations. As a result, studies vary widely in sampling approaches (vacuum dust or wipes), areas in the home that are selected for sampling, and the particle size fraction analysed [20]. Such methodological differences may be unavoidable but they can make it difficult to compare reported findings. The majority of studies use concentration metrics (e.g., mg/kg) to measure contaminants in dust, but dust loading metrics (e.g., mg/m^2^) are also important for exposure estimates [20,22,23,24,25]. Some studies that compared both metrics have reported a “dilution effect” caused by high dust loading, that is, dust concentrations of certain synthetic organics, such as phthalates and flame retardants, may decrease as dust loading increases [18,19,25,26]. Not all organics display an equivalent dilution effect, even under the same dust loading conditions [18], and Jílková et al. [20] did not observe this relationship for any flame retardant that they studied.

A key disparity amongst house dust studies is the particle size fraction selected for analysis of target contaminants. No size fractionation occurs in standard wipe sampling protocols, with the result that studies aiming to compare wipe and vacuum results tend to use relatively large particle size fractions (<300 or <500 μm) to facilitate direct comparisons [19,20,22,25]. Generally, finer particle size fractions are preferred for exposure assessments based on two main considerations: (1) greater adherence of finer particles onto skin increases their relevance for assessing ingestion and dermal exposures, and (2) greater surface area to mass ratio of finer particles enhances contaminant adsorption [2,16,27]. For assessing exposure through dust ingestion, US-EPA [28] recommends size fractions that adhere to hands (<150 μm). This recommendation is consistent with the conclusion by Cao et al. [29] that risk assessments should focus on the <100 μm particle size fraction of indoor dust, based on observations that concentrations of toxic chemicals in dust commonly increase as particle size decreases. Additionally, finer dust fractions have the advantage of greater analytical homogeneity [16,22,30] and greater relevance for estimating inhalation exposures to resuspended dust [7,31].

Questionnaires administered in house dust studies vary according to the purpose, scale and design of the study, and (depending on jurisdiction) ethical, legal and privacy constraints. Large randomized dust studies, aimed at quantifying population-scale exposures to chemicals, tend to document basic characteristics of each household, such as house age, building style, socioeconomic status, smoking behaviour, types of flooring and heating fuel [13,17,32,33]. Hypothesis-driven studies, which tend to address one contaminant or chemical class at a time (such as lead or flame retardants), require detailed observations of potential sources, e.g., indoor paint and other interior decoration materials, room-by-room variations in furniture, and use of electronic devices [25,34,35]. Such hypothesis-driven approaches are the basis for intervention studies designed to identify and mitigate specific indoor sources of target substances. Information about local outdoor sources and occupations helps to identify contaminants from vehicular traffic and nearby industry such as mining and smelting operations [23,24,36], and contaminants brought home from the workplace [2], including take-home exposures to pesticides from agricultural fields [37,38]. Questionnaires that accompany house dust studies also vary depending on whether researchers administer the questionnaire during the home visit, as in the Canadian House Dust Study [17,22,39], or if participants complete the questionnaire themselves, as in recent Citizen Science approaches [40,41,42].

The present study examines relationships amongst house characteristics observed in the Canadian House Dust Study (CHDS) and uses these relationships to assist in the interpretation of house dust chemistry. The CHDS was a population-based study designed to establish national baseline concentrations of chemicals in settled dust from urban, single-family houses [22,39]. The CHDS was not designed to identify specific indoor or outdoor sources, but basic characteristics of each household were documented to assist in identifying general trends. Composite (whole-house) vacuum dust samples were collected from 13 cities during the winter season of 2007 to 2010 (incl.) and analysed quantitatively for organic and inorganic contaminants [17,22,39,43,44,45,46,47,48,49,50,51,52,53,54,55]. This study uses Spearman rank analysis to identify relationships between house characteristics and 59 elements (metals and metalloids), and >200 synthetic organics, including phthalates; bisphenol A (BPA) and BPA analogues; organophosphate esters (OPEs); halogenated flame retardants including polybrominated diphenyl ethers (BDEs); octylphenol and nonylphenol ethoxylates; aryl and alkyl-aryl phosphates; bactericides (parabens); pesticides (e.g., permethrin, organochlorines); and synthetic musks. This paper focuses first on chemical concentrations in house dust, to assist in interpreting potential sources, and second on dust loading, to assist in identifying key exposure factors.

## 2. Methods

### 2.1. Study Design and Questionnaire Administration

The Canadian House Dust Study (CHDS) was designed to obtain a stratified random sample of detached single-family dwellings across 13 Canadian cities having a population greater than 100,000 [22,39]. Health Canada’s Research Ethics Board (REB), which approved the CHDS, limited the household visits to two hours, during which time the technicians completed all sampling and documented the house characteristics by taking measurements and administering a questionnaire (Table 1). The CHDS questionnaire was short due to the time constraint and limits on content (only questions directly related to dust composition were permitted). The sampling technicians documented descriptions of the floor surface, and measured the dimensions of the sampled area, for calculations of dust mass loading, and %carpet, %vinyl and %hardwood in each home (Table 1). The technicians also documented the time elapsed since the last cleaning, required to calculate the dust loading rate (Table 1). The number of children in the home (#children, Table 1) refers to children under 12 years of age. Many homes used a combination of heating styles; for example, wood fuel was commonly used in combination with other fuels, and therefore results related to wood fuel refer to “any fuel plus wood” (unless stated otherwise). Additional questions, not discussed in this paper, were specifically related to the interpretation of dust lead (Pb) sources and speciation, including hobbies, occupations, and recent renovations, as described previously [39,47,56].

### 2.2. Sample Collection, Preparation and Analysis

Settled dust samples were collected from 1025 randomly selected homes by trained technicians, during the winter seasons from January 2007 to March 2010 (incl.), as described previously [22,39]. The CHDS vacuum sampling protocol was designed to collect a consistent composite (whole-house) sample of readily accessible “fresh dust” of known age, from dry floor surfaces of known dimensions, to provide a complete set of elemental concentration, load, and loading rate data [22,39]. Damp areas, such as bathrooms and unfinished basements, were avoided to preserve sample integrity. Where available, a household vacuum sample was also collected from the participant’s vacuum system used for their regular cleaning activities, for comparison with the fresh dust sample. All dust samples were air-dried and sieved to fine (<80 μm) and coarse (80–300 μm) size fractions, and weights of each fraction were documented for loading calculations [22]. Chemical analyses were performed on the <80 μm fraction, and all chemical loading calculations in the present paper used the <80 μm fraction. Data summaries for all of the substances included in this paper are provided in the Supplementary Information (SI-6 to SI-16), and details of the analytical methods were published previously. Analytical approaches were developed or optimized, in Health Canada laboratories, to provide quantitative determinations of the synthetic organic compounds [43,44,45,46,48,49,50,51,52,54]. Total element concentrations were determined using Inductively Coupled Plasma Mass Spectrometry (ICP–MS), Inductively Coupled Plasma Optical Emission Spectrometry (ICP-OES), Instrumental Neutron Activation Analysis (INAA), or (in the case of mercury) direct solid sample analysis using atomic absorption spectrometry [17,22,47,55]. The six larger datasets (642 to 1025 homes) used all samples that were available at the time of analysis, whereas the five smaller datasets (119 to 263 homes) were based on representative subsampling of all 13 cities. See original publications for details of sample selection, analytical methods and quality assurance.

### 2.3. Statistical Analysis

Datasets were stored in an Oracle Database (version 12c), and extracted for statistical analysis using a combination of SPSS statistical software (version 21) and Microsoft Excel (version 2016). Nonparametric statistics were used for correlating concentration data with questionnaire data (Spearman Rank, Wilcoxon Rank, and Mann–Whitney U-tests) because frequency distributions of most chemical concentration datasets (μg/g) were neither normal nor lognormal (according to Shapiro–Wilk and/or Kolmogorov–Smirnov analyses). In this paper, “*r*” and “*p*” values refer to Spearman *rho* and significance, respectively, unless indicated otherwise. Significance of all Spearman Rank results for two-category questionnaire data (yes/no responses) was confirmed using Mann–Whitney U-tests. The loading datasets were generally lognormally distributed, allowing both nonparametric (Spearman rank) and parametric tests (Pearson correlations) to be used for analyses of loading results. See original publications for comparisons of technician-collected fresh dust (FD) samples and household vacuum (HV) samples for all substances. In the present study, the FD datasets were preferred for Spearman rank analyses, if available. Rasmussen et al. [17] evaluated the effect of using HV data in Spearman rank analyses (instead of FD data), for correlations between chemicals and house characteristics, and showed that HV data indicated similar trends as FD data, except for the loss or lowering of the statistical significance of associations between HV and questionnaire data (for some elements). Flooring was treated as continuous data (%carpet, %hardwood, %vinyl) with the exception of the loading calculations (detailed in Section 3.5), for which %carpet was grouped into quartiles of CHDS homes. The lowest quartile included homes with 0–18% carpet cover; next quartile 18–47%; next quartile 47–75%; and the top quartile was characterized by 75–100% carpet cover.

## 3. Results and Discussion

In the first part of this study, relationships between house characteristics and chemical concentrations in dust are examined using Spearman rank correlation analyses (summarized in Supplementary Information SI-1 and SI-2). This is an exercise in “pattern recognition”, which has the goal of identifying trends that can point to possible sources of chemicals. The second part of this study focuses on chemical loading (in contrast to chemical concentration), in relation to house characteristics. For some chemicals (e.g., lead), dust loading metrics may predict chemical body burden (hence exposure) better than chemical concentrations in dust [25,57]. Previous analysis of CHDS results for metals showed that dust mass was the main influence on metal loading: for example, multi-variate analysis of cadmium (Cd) showed that the Cd loading rate was influenced 68% by dust loading and only 32% by the Cd concentration of dust (*p* < 0.001) [22]. It was concluded that, while concentration data were useful for indicating the presence of metal sources in the home, dust load had an overriding influence on metal load and metal loading rates [22]. The present study will compare dust loading and concentration metrics for organic compounds in the context of two important exposure factors: carpets and proximity to industry.

### 3.1. Pattern Recognition Using Spearman Rank Analysis

Pattern recognition is a starting point for developing testable hypotheses for future investigation, whether experimentally or with future house dust monitoring. This exercise also serves to inform and improve survey questions for future studies. For example, CHDS questions about smoking behaviour focused solely on the use of cigarettes and cigars/pipes (Table 1). After the analytical results were available, statistical analysis indicated significantly elevated concentrations of rare earth elements (REEs) in settled dust of smokers’ homes [17]. The observed enrichment of REE was attributed to emissions from cigarette lighter flints made of the REE alloy “mischmetal” [17], an interpretation that was supported by independent particle characterisation research [58] and subsequently investigated as a potential inhalation exposure pathway [59]. Based on this outcome, future studies may consider documenting information about cigarette lighters, but at the time the CHDS was designed, there was no evidence that lighter flints contributed REE to house dust. This example of identifying an unexpected trend, by combining indoor dust chemistry results with questionnaire data, shows the value of applying a “pattern recognition” approach to population-scale house dust studies.

The classes of chemical substances measured in CHDS samples (published to date) are listed in Table 2, with references to source publications that provide details of the analytical methods, limits of detection (LODs), and detection frequencies. The acronym “BDE” is used in this paper to refer to the studied polybrominated diphenyl esters, which are all BDE compounds (SI-6). Also listed in Table 2 are the house characteristics that show significant correlations with concentrations of each class in settled house dust, based on Spearman rank analyses from the present study (summarized in SI-1 and SI-2). This is the first study of chemical correlations with house characteristics for the organic classes in the CHDS; some relationships have been discussed previously for selected metal(loid)s [17,22,39]. There are three house characteristics in Table 2 for which correlations with chemical substances may be positive or negative (details in SI-1 and SI-2): construction date, municipal zone, and dust loading. To clarify, a negative correlation between the concentration of a substance and the construction date means that the concentration increases with house age (because construction date increases as house age decreases; Table 1). A negative correlation between substance concentration and municipal zone means the concentration increases as the home location approaches the urban core (numerical code for municipal zone increases from urban core to suburban fringe locations; Table 1). Finally, a negative correlation between the concentration of a substance and the dust-loading rate indicates that increased dust loading has diluted the substance concentration (discussed later). Apart from these three characteristics, other negative substance correlations generally mirror correlations with related house characteristics, as discussed previously (Table 5 in reference [17]) and have been deleted from SI-1 and SI-2 to avoid confusion.

#### Limitations and Challenges

Further testing will be needed to confirm any source–receptor relationships suggested by the trends and patterns identified in this study. Several challenges are associated with interpreting relationships between chemical concentrations in house dust and house characteristics. The first challenge is identifying multiple overlapping influences on substance concentrations. To assist in identifying overlapping influences, all relationships between house characteristics in Table 1 are provided in SI-3. The organophosphate esters (OPEs) provide an example: Table 2 indicates that both %vinyl (i.e., the percentage of sampled floor surface covered with vinyl flooring) and the use of room deodorizers (i.e., air fresheners) correlate with OPE concentrations. Tables SI-1b and SI-1d indicate that the same OPE compound (TBOEP) correlates with both house characteristics, and furthermore, SI-3 indicates that these two house characteristics also correlate with each other (*p* < 0.01). As TBOEP is commonly used in vinyl resins and floor polishes [60], the significant correlation between TBOEP concentrations in dust and vinyl flooring (*p* < 0.05; SI-1b) is consistent with its known properties and applications. However, the same OPE compound (TBOEP) actually shows a more significant correlation with use of deodorizers (*p* < 0.01; SI-1d). The latter relationship is more difficult to assess because information on air freshener ingredients, including potential flame-retardant additives, tends to be proprietary [61]. Thus, the preliminary observation of a significant relationship between deodorizer use and OPE concentrations in house dust (SI-3) may be regarded as a hypothetical source that needs to be tested experimentally. Nevertheless, the point remains that to effectively assess the %vinyl–OPE relationship reported in Table 2, it would be prudent to consider the potential overlapping influence of related house characteristics (such as room deodorizers) on OPE concentrations. In summary, the relationships summarized in Table 2 should be regarded as uncensored Spearman rank results (SI-1 and SI-2), potentially influenced by inter-related house characteristics (SI-3).

Another challenge is that relationships between house characteristics can also be a source of artefacts or spurious correlations unless they are recognized. The use of radiators for heat distribution provides an example. Only 2% of CHDS homes use radiators (*n* = 20; see SI-4), and the negative correlation between radiators and construction date (SI-3) indicates that radiators tend to be a feature of older homes. The Supplementary File SI-1a shows a weak but significant correlation between radiators and obsolete pesticides (such as DDT; r < 0.2; *p* < 0.01). Such a relationship would be difficult to interpret, unless placed into the context of the correlation between construction date and DDT (r = −0.3 to −0.4; *p* < 0.01, SI-1b), discussed later in this paper. Based on the greater strength of the correlation between house age and DDT, it follows that the relationship between radiators and DDT (SI-1a) may be an artefact of the relationship between radiators and house age (SI-3). Other potential artefacts may be identified by referencing the uncensored Spearman rank results in the Supplementary Information (SI-3). It is important to be aware of these potential artefacts because they underscore the need to identify and avoid spurious correlations when interpreting questionnaire data.

A further challenge is associated with interpreting results of smaller house dust studies (e.g., fewer than 300 homes). Table 2 differentiates between the six larger datasets (642 to 1025 homes) and the five smaller datasets (119 to 263 homes). An important advantage of the larger datasets is that they capture a wider variety of homes, and display a greater number of significant correlations between substances and house characteristics (as shown in Table 2), which facilitates source interpretations. For example, the large OPE dataset (*n* = 814 homes; Table 2) displays significant correlations between OPE concentrations and the number of children in the home (#children), which would point to children’s products as a potential source of OPEs. This interpretation is consistent with the recent Canadian CHILD cohort study [60] that linked children’s products with elevated concentrations of OPEs in house dust. Phthalates have also been linked to children’s products [62], but in contrast to the OPE results, no correlations between phthalate concentrations and the number of children were observed in the present study (Table 2; SI-2c). The reason for the absence of this correlation is unknown: it may be due to the ubiquitous presence of phthalates in all homes, with or without children, or it may be attributable to the smaller size of the phthalate dataset (*n* = 126 homes). In the case of studies with a random sampling design such as the CHDS, these examples illustrate the benefit of investing resources in representative studies of sufficient sample size to facilitate source interpretations.

### 3.2. Chemical Concentrations in Relation to Building Characteristics

Significant correlations exist amongst certain characteristics of the CHDS homes, which are important to examine before linking dust concentration data with any single characteristic. In this context, Table 3 lists some key relationships revealed by Spearman rank analysis of the questionnaire data (see SI-3 for all results), which may be negative or positive depending on the assigned numerical coding (Table 1). For example, Table 3 indicates that newer homes tend to have attached garages, based on the observed positive association between construction date (which increases as house age decreases) and the presence of an attached garage (no = 0; yes = 1; Table 1). Similarly, the positive association between construction date and municipal zone (Table 3) reflects the tendency for newer homes to be located in suburbs, because the numerical code for municipal zone increases from the urban core to the suburban fringe (Table 1). Negative correlations with construction date in Table 3 indicate that hardwood flooring tends to be associated with older homes in the CHDS, as is the use of oil as a heating fuel and radiators for heat distribution. The positive association between dust loading rate and proximity to industry (defined broadly as <2 km from any industry) was discussed previously [22] and will be explored later in this paper. It is significant for the purpose of this paper to recognize that there is no association between proximity to industry and construction date of CHDS homes, or between proximity to industry and municipal zone (SI-3).

#### 3.2.1. Trends Associated with Increasing House Age and Proximity to the Urban Core

Construction dates of the 1025 participating CHDS homes ranged from 1832 to 2009 (mean ± sd = 1965 ± 27). Selected contaminants that are associated with increasing house age and/or proximity to the urban core (i.e., a negatively correlated with construction date and/or municipal zone) are compiled in Table 4 (see SI-1 and SI-2 for all results). As shown in Table 3, hardwood flooring, oil fuel, wood fuel, and radiators are associated with increasing house age, while electric heat and baseboards are associated with proximity to the urban core. Therefore, these related characteristics and their associated contaminants have been included in Table 4 to assist in identifying trends.

Contaminants such as lead (Pb) that display increasing concentrations in dust as home locations approach the urban core (Municipal Zone; Table 4) likely have outdoor sources as well as indoor sources [39,63]. Of all the house characteristics, house age displays some of the strongest correlations with contaminants in dust (Table 4), including dozens of metal(loid)s. These trends are consistent with other studies reporting that dust concentrations of certain metal(loid)s increase with house age [36,41]. The negative correlations between potentially toxic metal(loid)s and construction date, including Pb, mercury (Hg), cadmium (Cd) and cobalt (Co), reflect their declining use in building materials, pigments, coatings, plastics and consumer products [55,64,65,66], due to voluntary initiatives and/or regulatory restrictions.

While Table 4 captures the general trend of the declining usage of legacy metals (reflected by negative correlations with construction date), the CHDS was not designed to identify specific sources such as Pb-based paints or Hg-containing fluorescent lamps. However, the side-by-side positioning of related house characteristics in Table 4 enables chemical trends to emerge that shed light on possible indoor sources. In particular, hardwood flooring emerges as a key indoor characteristic that correlates with inorganic and organic contaminants in house dust (Table 4; SI-1; SI-2). Hardwood flooring is a manufactured wood product that incorporates a variety of adhesives, extenders, fillers, and binders along with plywood in the finished product, plus significant quantities of pigments and coatings [17,67]. All of these synthetic components of hardwood flooring contain a wide range of metals, flame retardants and phthalates [67]. The US Consumer Product Safety Commission (CPSC) [67] tested a variety of manufactured wood products for 8 metals and 10 phthalates and found the highest concentrations in hardwood flooring and particleboard.

The observed association of diisobutyl phthalate (DIBP) with hardwood flooring in the present study (Table 4) is consistent with the above findings by CPSC [67], and also with its use in hardwood varnishes [68]. Because DIBP has similar properties as dibutyl phthalate (DBP), it tends to be used interchangeably with DBP [69]. The strong association of DBP with house age (Table 4) suggests that other furnishings and building materials may be considered as potential indoor sources. A more detailed study design would be required, however, to identify other manufactured wood sources, besides hardwood, such as wood panelling and furniture. The association of OPEs with older homes and with hardwood flooring in particular (such as EDHPP, TBOEP and TPHP in Table 4) is consistent with the use of these chemicals in polymers, adhesives, sealants, coating products and floor polishes [70,71]. In contrast to the OPE class, it is notable that the halogenated flame retardants, including BDEs, did not display significant associations with these characteristics of older homes (SI-1, SI-2).

As %hardwood, radiators, and oil heat are all associated with increasing house age (Table 3) it is important to consider overlapping influences of these related house characteristics. For example, the association between Pb in dust and radiators (*p* < 0.01; Table 4) may be attributable to deteriorating Pb paint on radiators, but also could be influenced by the relatively stronger associations between Pb and house age (Table 4) and between radiators and house age (Table 3). Not included in Table 4 is the significant association between nonylphenols and radiators (*p* < 0.01; SI-1a), which may reflect their use to inhibit corrosion and scale formation [72]. The influence of wood fuel on dust chemistry (Table 4) is discussed later in this paper.

TPHP is used as a flame retardant in electronics and thermoplastics [71], which may explain its occurrence in the electrical heat and baseboard categories (Table 4). The germanium (Ge) trends across Table 4 are quite compelling: it is notable that Ge concentrations in dust actually correlate more strongly with baseboard heaters and with hardwood floors than with house age (Table 4). The associations of Ge with these specific indoor sources are consistent with the use of Ge both as a semi-conductor and as a catalyst in the manufacture of plastics [64]. Similarly, tin (Sn), which appears together with Ge in both hardwood and baseboard heat categories (Table 4), is used both in the manufacture of plastics (as organotin) and in electrical applications (solders and fluxes). The results in Table 4 suggest that both applications are present as indoor sources of Sn and Ge, especially in older homes.

It is more difficult to interpret the association of thallium (Tl) with hardwood, and with house age in general (Table 4), due to a lack of detailed information on end-uses of thallium [64]. Apart from its uses as an insecticide and rodenticide, Tl is used for the synthesis of organic compounds [64,73]. Thus, while it is possible that Tl is present as a wood preservative, it is also possible that Tl occurs with other metals in the synthetic components of hardwood flooring. In summary, hardwood flooring as a source of metals and synthetic organics in house dust may arise from treatment of the wood itself, from components used in the manufacture of hardwood flooring, from the stains used to colour the wood, and/or from varnishes and other coating materials. These appear to be released during the normal wear and tear of hardwood floors and subsequently accumulate in dust, as suggested by the many significant associations between %hardwood and dust contaminants (Table 4; SI-1; SI-2).

Synthetic musks, which are semi-volatile compounds used in personal care products, are known to be very persistent in environmental media [74]. Internationally, the use of nitro musks, including musk xylene (MX) and musk ketone (MK), has declined since the 1980s because of voluntary bans and/or restrictions imposed in various jurisdictions [75,76,77]. This decline is reflected in the present study (Table 4) by the significant correlations between musk concentrations and house age and associated characteristics. Also significant are the correlations between house age and concentrations of the obsolete dichlorodiphenyldichloroethane compounds (DDTs and their transformation products DDE and DDD) in house dust. Further analysis using the sum of these compounds (ΣDDT) indicated detection in 69% of the CHDS homes (predominantly in homes built before 1975), which is consistent with detection rates of 70–78% reported for DDT in house dust collected in German cities in the 1990s [2]. The presence of obsolete organochlorine compounds in indoor settings, including Aldrin and DDT, has been attributed to their historical use as wood preservatives, textile and carpet treatments, and insecticides and herbicides for lawn maintenance [78,79]. The present study shows that concentrations of DDT, DDD and DDE in dust are significantly associated with hardwood flooring (Table 4) but not with %carpet (SI-1b), pointing towards treated wood as a more likely source than carpets in CHDS homes. The significant correlation between ΣDDT and hardwood floors occurs only in the subset of homes built in 1975 or earlier (*p* < 0.001) and not in the post-1975 subset (*p* = 0.60). The presence of ΣDDT in dust samples collected in the CHDS (2007 -2010) over > 35 years since DDT was banned demonstrates the persistence of these compounds in indoor environments.

#### 3.2.2. Trends Associated with Increasing Construction Date and Proximity to Suburban Fringe

Table 5 compiles selected substances and house characteristics that positively correlate with increasing construction date (i.e., decreasing house age) or increasing municipal zone (i.e., home location approaching suburban fringe). Construction date correlates positively (at *p* < 0.01) with municipal zone, attached garages and fireplaces, as listed in Table 3, whereas municipal zone correlates positively with use of forced air (*p* < 0.01), gas heating (*p* < 0.01) and stove heaters (*p* < 0.05; SI-3). In addition to the positive correlation between %carpet and construction date shown in Table 3, %carpet also correlates positively with municipal zone, attached garages, forced air, gas heat and fireplaces (all at *p* < 0.01; SI-3).

In contrast to Table 4, which features decreasing concentrations of legacy contaminants with increasing construction date, a number of “emerging” and “alternative” synthetic organics appear in Table 5, many of which are used in heat-resistant building materials, including alloys, rubber and plastics. For example, bisphenol M (BPM) is used to make heat-resistant polymerized resins [80]. This BPA analogue compound correlates only with forced air in the present study, with an unusually high rho value (r = 0.4 to 0.5; *p* < 0.01; Table 5) considering the relatively small dataset (*n* = 119; Table 2). The compound 4-tertoctylphenol (4-tert-op) is used in the formulation of rubber and heat-cured resins for insulating electric windings in motors and transformers [81] and is also significantly associated with forced air (Table 5). Nonylphenol ethoxylates such as NP2EO (associated with gas heating in Table 5) can be substituted by 4-tert-op in most alkylphenol ethoxylate uses [81].

Several metal(loid)s are strongly associated with forced air heat distribution in Table 5, which may arise from heat-resistant alloys used in residential furnace systems. For example, molybdenum (Mo) and hafnium (Hf) are the main components of high-temperature carbide alloys [82] and are added to polymetallic alloys to improve thermal stability and tensile strength [83]. Organotellurium (Te) is used to improve the thermal stability of rubber, polyethylene and polypropylene [84]. Boron (B) is used in flame retardants and insulation [85], and thermal properties of B are similar to those of Hf [86]. Blount [87] describes how metal(loid)s that react with phosphorus or halogen compounds to form a salt are suitable for the manufacture of flame-retardant polyurethane products (e.g., coatings, thermal insulation), including the alkali metals sodium (Na), potassium (K), lithium (Li), rubidium (Rb) and cesium (Cs) and the alkaline earth metals calcium (Ca), strontium (Sr), magnesium (Mg) and barium (Ba). The observation that these elements correlate with a number of heating styles, including forced air and stoves (Table 5 and Table 6, SI-1a), is consistent with their application in flame-retardant materials. Thus, it appears very likely that many heat-resistant compounds are being released from components of the residential heating system rather than from the fuel itself.

In general, it is difficult to determine whether fuel or the heat distribution system is the main source of metal(loid)s in dust. Of all the elements associated with stove heating, Mn displays the strongest correlation (Table 5). As Mn also correlates with wood fuel (*p* < 0.01; SI-1a, Table 4), and is reportedly enriched in ash arising from natural biomass combustion [88], it appears likely that particle-bound Mn is released from wood combustion. Similarly, the use of wood fuel for heating Australian homes was associated with increased Mn concentrations in vacuum dust samples [40]. However, it is notable that Mn is used as a drier for coatings used in stoving (at concentrations of 0.02–0.05%; [89]) and as a dark colourant in ceramic glaze [90]. In addition, stoves used for home heating are typically made from cast iron and plate steel and Mn is used as an alloy in steel [64]. Therefore, a combination of emissions from the stove itself and the biomass being burned in the stove could account for the relatively strong correlation between Mn and stove heating (Table 5). Likewise, it is difficult to identify the precise source of tantalum (Ta) in heating systems. In addition to the correlation between tantalum (Ta) and use of gas fuel and fireplaces (*p* < 0.01; Table 5), Ta also correlates with wood fuel (*p* < 0.05; SI-1a). Since Ta is also used in high temperature applications (e.g., heat shields and thermocouple shields; [64]), it appears possible that Ta could be released from fireplace components, rather than from the fuel being burned. Similarly, the correlation between the synthetic compound isodecyl diphenyl phosphate (IDDPHP) and fireplaces also may arise from the fireplace itself rather than from the fuel, because IDDPHP has various uses as a flame retardant, plasticizer, stabilizer, and lubricant [91]. It is feasible, however, that the association of parabens (bactericides) with fireplaces (Table 5) may indicate treatment of manufactured biofuels, such as firestarter logs made of sawdust and wax. Some firestarter logs also contain metals that are added to create colours when burned, which can make them hazardous for pets if ingested [92].

The positive associations in Table 5 between magnesium (Mg) concentrations in dust and municipal zone (i.e., higher concentrations in suburban areas) may reflect the recent increased use of Mg in building materials. Building boards made with magnesium oxide are a relatively new alternative to traditional sheeting materials such as calcium sulphate (gypsum) plasterboard, fibre-cement board and plywood [93]. The association of forced air with Mg concentrations in dust (Table 5) may reflect the use of Mg as a refractory material in furnace linings [64] or it may indicate that forced air distribution systems redistribute particle-bound chemicals from building materials such as magnesium oxide or gypsum boards, or from a variety of other sources unrelated to the furnace itself. The possibility that particles are redistributed within the home by forced air systems is supported by the observed correlation between forced air and dust loading (*p* < 0.01; SI-3).

Table 5 displays several building characteristics that display significant positive correlations with halogenated flame retardants, including construction date, attached garages, carpets, gas heating, and forced air distribution systems (in contrast with Table 4). Hexabromo biphenyl (HBB), which is considered a “novel” brominated flame retardant [94], is associated with both forced air systems and stove heating in the present study (Table 5). This observation is consistent with the known application of brominated flame retardants in kitchen hoods, pipes and insulation [95]. Similarly, the association of organophosphate flame retardants/plasticizers with heating systems in Table 5 is probably related to their use in fireproof resins and plastic coatings. It is notable that IDDPHP, also an “emerging substance”, is the only organophosphate flame retardant to show a positive correlation with construction date and related characteristics in Table 5. This is a significant difference from Table 4, which showed that numerous organophosphates displayed significant negative correlations with construction date and related characteristics.

The association of BPA, and its analogue bisphenol S (BPS), with %carpet (Table 5), appears to be consistent with the use of plasticizers in carpet backing [8]. Table 5 indicates that %carpet cover is correlated with halogenated flame retardants (including BDEs) and bromine (Br), which is considered a marker for brominated flame retardants [96]. These correlations are consistent with the use of flame retardants in the manufacture of carpets and carpet padding [8,97]. The association of boron (B) with %carpet would need further investigation to determine if B is released from carpets, or if it is applied as a carpet cleaner, or if particle-bound B accumulates in carpet dust from other household sources. In this context, Haines et al. [8] noted that it is important to distinguish whether carpets are acting as a repository for pollutants, or as a primary source. The pattern that emerges from Table 4 and Table 5 (and SI-1b) indicates that halogenated flame retardants (including BDEs) tend to correlate with carpets but not with hard floor surfaces, in contrast with OPEs and ∑DDT, which tend to correlate with hard floors but not carpets. Percy et al. [25] also found higher dust concentrations of OPEs associated with hard floors (83% of which were wood floors) than with carpets. This strong contrast in the influence of carpets versus hard surfaces on dust chemistry suggests that the materials themselves are acting as primary pollutant sources.

It is notable that only three metal(loid)s (B, Br and Hf) display a positive correlation with construction date (Table 5), in contrast with the large number of metal(loid)s that display a negative correlation with construction date (Table 4). As discussed above, the trend for Br appears to reflect the use of brominated flame retardants and the Hf trend appears to reflect its use in heat-resistant alloys, whereas B has numerous household uses, including insect control and being a flame retardant, cleaning agent, and wood preservative, and it is used in a wide variety of house construction materials, including flooring, wall materials/drywall, wall-to-wall carpets, and insulation [85].

#### 3.2.3. Associations between Metal(loid)s and Flame Retardants in Dust

Residential sources of metal(loid)s are difficult to identify using questionnaires because they may arise from both outdoor and indoor environments, and because they occur in so many building materials and/or common consumer products that they tend to be ubiquitous in house dust. For example, Zn is enriched in indoor dust in the CHDS and many other studies [22,27,98,99], but correlates with only two house characteristics (Table 4 only). Table 6 shows that Zn and many other metal(loid)s are positively correlated (*p* < 0.05) with flame retardants in CHDS homes. Consistent with the claims in the patent by Blount [87], Zn displays correlations with three OPEs, one BDE, and four non-BDE halogenated flame retardants (Table 6). Note that the use of Zn in flame-retardant formulations would not be captured using a questionnaire. See SI-17 for further discussion of chemicals (Zn and triclosan) and building materials (vinyl) that displayed few associations with house characteristics.

Previously, Table 4 and Table 5 indicated which flame-retardant compounds were associated with specific building characteristics (floor types and heating styles). Further to those observations, Table 6 shows which metal(loid)s are correlated with each of those particular flame retardants. The correlation of Br with each BDE in Table 6 reflects the presence of Br as a molecular constituent of brominated flame retardants. In contrast, antimony (Sb), which is an inorganic flame retardant, may correlate with organic flame retardants in Table 6 because their similar applications have resulted in their co-occurrence. Likewise, correlations between dust concentrations of TPHP, Ge and Sn (Table 6) likely reflect their co-occurrence in the synthetic components of hardwood flooring, and in electrical appliances such as baseboard heaters (Table 4), because all three substances are used in electrical applications and in the manufacture of plastics [64,71,100]. Similarly, Kefeni et al. [101] reported a significant positive correlation (*p* < 0.01) between summed trace metals and summed PBDE congeners in house dust (but not office dust), suggesting the formation of coordination bonds between PBDEs and metal cations [101].

### 3.3. Heating Fuels Versus Distribution Systems as Contaminant Sources

The above discussions noted the difficulty in determining whether it is the fuel or the heat distribution system that is the main source of contaminants associated with various styles of home heating (Table 4 and Table 5; SI-1a). Heating styles used in CHDS homes are summarized in SI-4. The use of fireproof resins and coatings in forced air furnace systems (including ductwork, wiring and motors) appears to contribute a variety of phosphorus and halogenated flame-retardant compounds to house dust (Table 5). The observation that metal(loid)s such as Br, Hf, Mo and Hg are significantly associated with these same flame retardants (Table 6) suggests the possibility that these contaminants may also arise from heat-resistant materials used in the furnace system rather than from the fuel itself.

**Figure 1:**
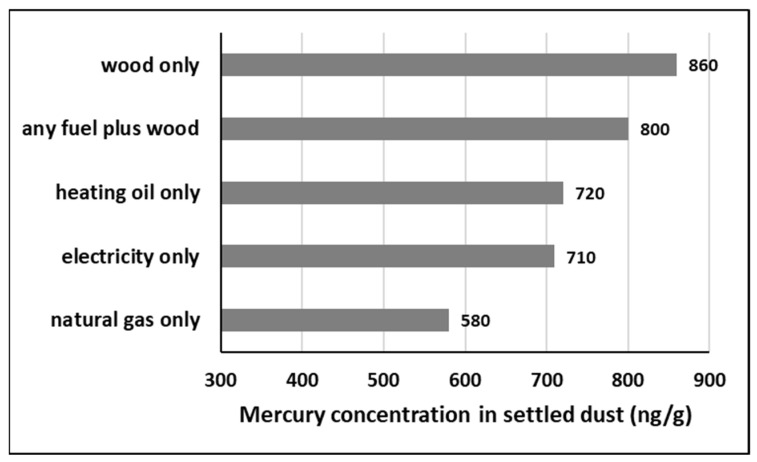
Influence of home heating fuel on mercury concentration in settled house dust (median values; ng/g). See SI-4 for summary of heating styles in CHDS homes.

This section attempts to identify the influence of fuel type, with emphasis on the common practice of using a combination of heating fuels. As each fuel type shows associations with a range of contaminants in house dust (SI-1 and SI-2), it follows that usage of a combination of fuels will exert an overlapping or additive influence on dust chemistry. To assess the influence of a given fuel type on metal(loid) concentrations in house dust, for example, natural gas, it is necessary to exclude homes that use another heating fuel in combination (i.e., electricity, heating oil, or wood). Mercury (Hg) is used here as the working example, as Hg concentrations display a lognormal distribution in the CHDS [55], allowing the use of parametric statistical tests to explore the influence of heating styles. Median Hg concentrations in house dust from homes using only one fuel type showed the following trend: wood > oil > electricity > natural gas (Figure 1), suggesting that wood combustion exerts the greatest influence. This interpretation is consistent with experimental research showing that wood combustion is a source of oxidized mercury (mostly in the particulate phase) in northern climates in winter [102]. The median Hg concentration for the entire dataset (690 ng/g; *n* = 995; [55]) is exceeded by the medians for homes heated solely by wood (860 ng/g; *n* = 27), heating oil (720 ng/g; *n* = 32) or electricity (710 ng/g; *n* = 72). Homes heated solely by natural gas display the lowest median Hg concentrations in house dust (580 ng/g; *n* = 434).

A fifth category was added to Figure 1, to examine the subset of homes that burn wood in combination with any other fuel, because only a small number of homes are heated solely by wood fuel (SI-4). The wood fuel category in Table 3 and Table 4 included homes that used “any fuel plus wood”; the majority of these (281 out of 308 homes) burned wood to supplement other heating sources. The relatively elevated median value for the “any fuel plus wood” category (800 ng/g; *n* = 308) supports the above finding that wood combustion has a greater influence on dust Hg concentrations than any other heating fuel. An independent t-test used to compare Hg dust concentrations in homes heated with “natural gas only” (*n* = 434) against homes using “any fuel plus wood” (*n* = 308) confirmed a significant difference in the geomeans of the two categories (*p* < 0.001).

There is evidence that heat distribution systems can also influence Hg concentrations in dust. Specifically, Hg concentrations are lower in homes using forced air furnaces (median Hg = 599 ng/g; *n* = 485) than in homes using baseboard heaters (median Hg = 749 ng/g; *n* = 181). The results of a t-test indicated that the difference in geomeans between the two distribution systems is statistically significant (*p* = 0.024). Possible explanations include release from mercury contact thermostats, which were once common in baseboard heaters [103], and/or the use of Hg-containing latex paint on baseboard heaters [104]. High-temperature resins and coatings are another possibility, based on the observed association of Hg with three organic flame retardants (Table 6).

Finally, it is important to consider the influence of house age when interpreting correlations between heating styles and contaminants in house dust. For example, the significant negative relationship between Hg concentrations in house dust and construction date (r = −0.38; *p* < 0.01; Table 4) could be a confounding factor when interpreting the influence of oil fuel on dust Hg concentrations, as it is the oldest homes that use oil fuel (average construction date of 1947). The correlation between “any fuel plus wood” and construction date (r = −0.07; *p* < 0.05) is very weak compared to other correlations with construction date shown in Table 3, but needs to be considered as a potential influence when interpreting the correlations between wood fuel and dust contaminants in Table 4. In contrast, the average construction date of homes using wood fuel alone (1970) is close to that of homes using natural gas alone (1968). Therefore, house age may not be a significant confounding factor with respect to the above interpretation (Figure 1), regarding wood fuel having a greater influence on Hg concentrations in house dust than gas or any other fuel. It is feasible that some chemicals emitted from burning wood originate from uptake and bioaccumulation by trees [67,105,106,107]. Other chemical associations with wood fuel in Table 4 and the Supplemental Information (SI-1a, SI-2a) could arise from burning treated wood or recycled wood products.

### 3.4. Influence of Characteristics Related to Consumer Products

Table 7 compiles substance correlations with household characteristics related to consumer products, and indicates which characteristics are also related to each other (Table 7 footnote). The association of OPEs, especially TBOEP, with children in the home (#children; Table 7) is consistent with previous studies that have linked OPE concentrations in house dust with children’s foam products, such as crib mattresses and foam toys [60,108]. Table 7 also indicates that a large number of BDE compounds are significantly associated with children in the home. A recent study reported relatively high concentrations of a range of brominated flame retardants in new and second-hand toys [94]. Therefore, a possible explanation for the BDE correlations in Table 7 is that particles from toys and other children’s products contribute BDEs to house dust. BDEs and bromine (Br) correlate with the use of upholstery treatments (Table 7). As the use of upholstery treatments correlates positively with the number of children in the home (Table 7), these two characteristics may have an overlapping influence on BDE and Br concentrations in dust. Similarly, the alkylphenol ethoxylate compound 4-tert-octylphenol (4-tert-OP) that appears in Table 7 correlated both with children and with upholstery treatments. Since the main use of 4-tert-OP is rubber [81], these associations may reflect releases from rubber toys.

Boron (B), which is used in household laundry and cleaning products, detergents, soaps and bleaches [64], correlates with use of deodorizers and upholstery cleaners and number of children in the home (all at *p* < 0.01; Table 7). Bismuth (Bi) is an over-the-counter pharmaceutical used as an internal deodorizer for stools and flatulence [109]. Thus, the positive correlation between Bi in dust and the reported use of deodorizers (Table 7) suggests the possibility that Bi is an ingredient in air freshener products. As expected, several synthetic musks display relatively strong correlations between their concentrations in dust and reported use of room deodorizers (Table 7). Additionally, Table 7 indicates that indoor exposures to a wide variety of other synthetic organic compounds are significantly associated with the use of room deodorizers, including nonylphenols, octylphenol, halogenated flame retardants, OPEs, aryl and alkyl-aryl phosphates, and BPA. For example, EHDPP is one of the OPEs that correlates with room deodorizers (Table 7), and this compound is known to be an ingredient in air fresheners [70]. Based on their research showing that OPEs are widely detected in pregnant women, Ingle et al. [110,111] underscored the need to assess the prevalence of OPEs in common household and personal care products in which they may occur as flame retardants, anti-foaming agents or plasticizers. Steinemann [61] stressed the need for research into ingredients used in air fresheners in particular, pointing out the paradox that “products designed to improve the indoor environment can pose unintended and unknown risks”. The large number of contaminants correlated with room deodorizers in the present study (Table 7; SI-2d) supports these concerns.

Many of the same chemicals that correlate with deodorizers also correlate with candles (Table 7), which may be influenced by the relatively strong correlation between these two household characteristics (Table 3). The correlation between sodium (Na) in dust and the use of candles (Table 7) is consistent with a study of candle emissions [112], which showed that candle soot contained elevated concentrations of sodium salts. In general, metals may be added to candle wicks as hardeners or flame retardants, and to candle wax as pigments [112].

Metal(loid)s and pesticides are the only chemical classes that correlate with reported insecticide use (Table 7). As insecticide use correlates positively with municipal zone (SI-3), proximity to rural areas and related house characteristics may influence these associations. Permethrin is a widely available insecticide product in Canada, consistent with the observed positive correlation between reported use of insecticides and permethrin in dust (Table 7). Some of the metalloids that correlate with reported use of insecticides in Table 7 have known applications as pesticides, including tellurium (Te), which is used as a fungicide and germicide [64], thallium, which is used as a pesticide for rodents and insects, [113] and Mn [114]. 

Results for dogs were not included in Table 7 because only two organic compounds, a nonylphenol (*n*-NP) and an aryl phosphate (tBPDPP), displayed significant correlations with the presence of dogs (SI-1c, SI-2c). In contrast, many chemical classes displayed significant correlations with the number of cats in the home (#cats; Table 7). Dust concentrations of three isomers of tetrabromoethylcyclohexane (TBECH), two nonylphenols, a synthetic musk, four OPEs, and several DDT and DDD isomers were significantly associated with the number of cats (Table 7). A Swedish study [115] found that commercial cat food was a significant source of several flame retardants (BDEs and halogenated phenols) and organochlorines (DDT and DDE), possibly introduced during manufacturing. In addition to cat food as a possible source, the chemical correlations in Table 7 may also reflect their use in consumer products associated with cats. Recent studies have identified cats as sentinels of household exposures to OPE flame retardants [116,117] but the results in Table 7 suggest that products associated with cats actually introduce OPEs and other organic contaminants into the home. Although permethrin is widely used for dog products that control fleas and ticks, alternative insecticides have been recommended for cats, because of the toxicity of permethrin to cats [118]. This may explain, to some degree, the observed dissimilarity between chemical associations with cats versus dogs (SI-1c; SI-2c). That is, several chemical classes have been used as pesticides or biocides for companion animals [71,81], and therefore correlations of some chemicals with cats in Table 7 may reflect their presence as formulants in flea and tick powders, shampoos, and flea collars. In addition, the use of cat litter may provide an explanation for correlations with synthetic organic compounds used as scents and deodorizers (Table 7). Strong correlations between numbers of cats and uranium (U), thorium (Th) and aluminum (Al) concentrations in dust (Table 7) were previously attributed to U and Th impurities in bentonite clay used for cat litter [17]. Dust concentrations of a number of metal(loid)s that correlate with the presence of dogs and cats (Table 7; SI-1c) are likely sourced from pet foods and/or track-in of soil minerals [17].

Apart from synthetic musks and phosphates, the other smaller datasets yielded relatively few significant correlations relevant to Table 7 (SI-2c to SI-2d). One notable observation is the correlation between dust concentrations of diethyl phthalate (DEP) with cigarette smokers (*p* < 0.05; SI-2c), which is consistent with previous observations of DEP in the particulate phase of cigarette smoke [119]. Two BPA analogue compounds, bisphenol-B (BPB) and bisphenol-AP (BPAP), also show significant correlations with cigar/pipe smokers (*p* < 0.05 for both), and BPAP concentrations correlate with cigarette smokers (*p* < 0.01; SI-2c). Dust concentrations of certain metal(loid)s are also associated with cigarette smokers (SI-1c).

### 3.5. Chemical Loading in Relation to House Characteristics

**Figure 2:**
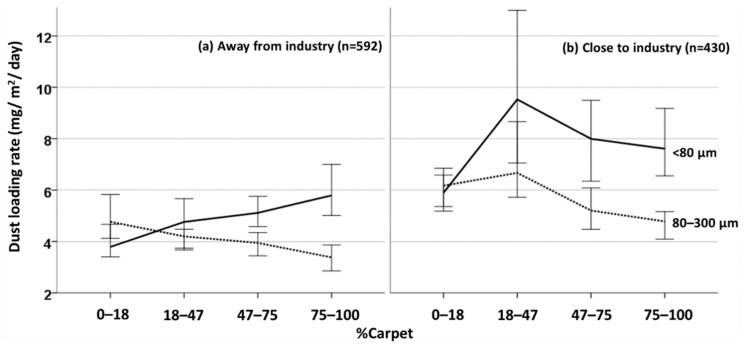
Influence of carpets on dust loading rates (mg/m^2^/day) in Canadian House Dust Study homes, located (**a**) more than 2 km from any industry and (**b**) within 2 km radius of any industry. Graphs show median values for two size fractions (<80 µm and 80–300 µm); error bars (75% CI) display variability of dust loading within each quartile. Details in SI-5.

Dust loading rates in CHDS homes increase significantly (*p* < 0.01) with proximity to industry, house age and increased carpet area (%carpet; Table 3). Other significant positive correlations with dust loading (at *p* < 0.01) include presence of smokers, forced air furnaces and numbers of dogs and cats (SI-3). Figure 2 illustrates the combined influence of two house characteristics that are unrelated to each other (*p* = 0.98)—%carpet and proximity to industry—on dust loading rates for two dust particle size fractions (<80 µm and 80–300 µm). See SI-5 for median values and sample sizes used to construct graphs in Figure 2. Overall, these graphs show that the dust loading rate of the finer fraction (i.e., <80 µm) increases incrementally as the percent carpet cover increases in homes away from industry (Figure 2a), whereas fine dust loading is higher in all homes close to industry, especially in homes with more than 18% carpet cover (Figure 2b). The conclusion drawn from these results is that homes with greater than 18% carpet cover that are located within 2 km of industry have the highest <80 µm dust load of any urban Canadian homes. These results are consistent with previous studies showing that mass loading of dust is greater in carpets than smooth floors [8,120,121]. The resuspension of dust following activity in carpeted areas is an important source of exposure to indoor pollutants, and has been linked to adverse health outcomes [121].

**Figure 3:**
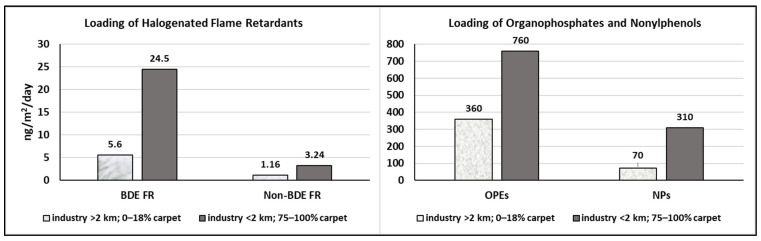
Summed exposures to four classes of co-occurring organic compounds in children’s homes: influence of carpet and proximity to industry. Median loading rates based on 80 µm dust size fraction (see Table 8 for significance and number of homes in each subset).

The influence of dust loading on childhood exposures to four classes of synthetic organics is illustrated in Figure 3, which examines subsets of homes in which children were living, for each end member in Figure 2. Each of these classes of co-occurring compounds, which include BDE flame retardants, replacement flame retardants (non-BDE halogenated compounds and OPEs) and nonylphenols, has relevance for the assessment of cumulative exposures to consumer product chemicals [6]. The term “summed exposure” is used in Figure 3 as the term “cumulative exposure” has specific connotations in some jurisdictions [122]. To provide context, the Supplementary Information (SI-6 to SI-15) lists median and 95th percentile loading rates for individual synthetic organic compounds. The bar graphs in Figure 3 demonstrate that children’s summed exposures to each class of compounds are likely to be elevated in heavily carpeted homes located near industrial zones, compared to homes located away from industry with little to no carpet cover (Figure 3), due to the strong influence of these two house characteristics on indoor dust loading.

Table 8 compares chemical loading versus concentration metrics for the same subsets of children’s homes shown in Figure 3. Differences in chemical loading between the end member subsets (<18% carpet/away from industry versus >75% carpet/close to industry) are significant (*p* < 0.05) for three chemical classes: ∑BDEs, ∑OPEs and ∑nonylphenols. (Significance is borderline for ∑non-BDE halogenated flame retardants; *p* = 0.1; Table 8). In contrast to loading metrics, chemical concentrations calculated using the same datasets (Table 8) are not significantly elevated in heavily carpeted homes located near industry. This difference between metrics echoes previous observations for metals, which showed that dust mass loading had a greater influence on metal loading than metal concentration [22]. Similarly, Table 8 demonstrates that dust mass loading has an overriding influence on loadings of synthetic organics.

Examination of the full OPE dataset (*n* = 816; SI-1c) shows that dust concentrations of three OPE compounds are significantly higher in homes located within 2 km of industry, notably TCEP and TCrP (both at *p* < 0.01) and TiBP (*p* < 0.05). (This trend did not appear in the small subsets of children’s homes in Table 8.) These results suggest an influence of external sources on house dust concentrations of certain OPEs, and may reflect usage of OPEs in such products as lubricants and hydraulic fluids, which are likely to be associated with industrial activity. Dimethyl phthalate (DMP), which is the only phthalate (and the only compound in the smaller datasets) that displays a significant increase in concentration with proximity to industry (SI-2c), is used in corrosion inhibitors and anti-scaling agents as well as in plastic and rubber products [123]. Concentrations of fourteen metal(oid)s also displayed a significant increase with proximity to industry (SI-1c), but further investigation would be needed to investigate these trends, as inorganic compounds are used in a wide variety of industrial applications, and “proximity to industry” is broadly defined as explained previously [22]. Evidence of the influence of home location on indoor exposures to contaminants, particularly in heavily carpeted homes (Figure 3, Table 8), provides support for studies addressing environmental equity concerns, such as those by Zota et al. [9] and Wan et al. [124].

### 3.6. Dilution or Enrichment Associated with Dust Loading

Table 2 indicates that most classes of chemicals displayed significant correlations with dust mass loading (only BDE flame retardants and musks did not). Details of the correlations between dust loading and chemical concentrations, including direction (positive or negative), are summarized in the Supplemental Information (SI-1d and SI-2d). These results show that variations in dust loading rates may or may not influence chemical concentration, and that the influence may be either negative (dilution) or positive (enrichment). Previous studies have shown that increased dust loading can dilute the concentrations of some substances in dust, and not others [18,19,25,26]. Similarly, the results of the present study (SI-1d and SI-2d) indicate that many substances display a negative correlation with dust loading (i.e., dilution effect), including several OPEs; certain metal(oids) such as Sb, Cr and Ni; parabens; two BPAA compounds; and numerous phthalates. Laboratory experiments designed by Bi et al. [18], to quantify direct source-to-dust transfer of phthalates, were consistent with these results in that they showed a decrease in DEHP concentrations under high dust loading conditions compared to low dust loading conditions. The authors’ interpretation was that increased dust loading increased the storage capacity of the dust for semi-volatile organics and slowed the direct transfer kinetics [18]. In the case of phthalates in CHDS homes (SI-2d), 11 compounds (including DEHP) displayed a significant negative correlation with dust loading rate (i.e., dilution). Of all the phthalates determined in the CHDS [49,52], DIDP displayed the strongest dilution effect from dust loading (r = −0.4 to −0.5; *p* < 0.01; SI-2d).

To our knowledge, this is the first study to show that many substances display positive correlations with dust loading, including one phthalate; one aryl phosphate; an octylphenol and two nonylphenols; organochlorine pesticides; numerous metal(oids) including potassium (K), cesium (Cs) and beryllium (Be); and two non-BDE halogenated flame retardants (see SI-1d and SI-2d). The one phthalate compound that displayed a positive correlation with dust loading (r = +0.2 to 0.3; *p* < 0.01; SI-2d) was dibutyl phthalate (DBP). DBP is present in consumer air fresheners at concentrations up to 4.5 ppm [125], and the present study displayed a positive relationship between reported use of room deodorizers and dust loading (significant at *p* < 0.05, in the subset of homes located > 2 km from industry). Therefore, it is possible that the positive correlation between dust loading and DBP concentrations may be related to its use in aerosol sprays. Loading rates are summarized in the Supplemental Information (SI-6 to SI-15) for all organic compounds determined in the < 80 µm size fraction of CHDS samples.

### 3.7. Implications of Using < 80 µm Particle Size Fraction for Dust Loading

Chemical loadings in the present study (Figure 3, Table 8; SI-6 to SI-15) were based on measurements of the <80 µm size fraction of settled house dust, consistent with previous recommendations to use fine dust fractions [29] for the determination of synthetic organics. It should be noted that chemical loadings calculated for the <80 µm size fraction (SI-6 to SI-15) average about 56% (by weight) of loadings calculated for the <300 µm size fraction (range 44–63 wt%; SI-5). Thus, for the purposes of comparison with wipe sampling studies, the <80 µm loadings in the present study should be approximately doubled, because the <300 µm size fraction is the fraction that yields comparable results to wipe sampling [22]. For estimating inhalation exposures to resuspended dust, previous CHDS measurements determined that about 50% of the mass of the <80µm fraction would be <10 µm [7], and about 50% of the mass of <10 µm fraction would be <2.5 µm [31].

The present study demonstrates the greater sensitivity of < 80 µm dust loading to percent carpet cover, compared to < 300 µm dust loading. In fact, the finer fraction (<80 µm expressed as wt%) displays a significant positive correlation with %carpet in CHDS homes (Pearson r = 0.40; *p* < 0.001; *n* = 1022; SI-5), whereas the opposite trend is observed for the coarser fraction (80–300 µm wt%; SI-5). Previous analysis of CHDS data showed that dust loading was not influenced by %carpet when the combined total mass of all particles <300µm was used to calculate loading (Figure 2 in reference [22]). The reason for selecting the < 300 µm size fraction previously was to facilitate the comparison of Pb loading values obtained using wipe sampling versus vacuum sampling in CHDS homes [21,22]. The CHDS sampling protocol required the technicians to deliberately use a “light touch” when vacuuming carpets in order to capture surface dust only (and avoid deep dust), to ensure that loading rates from carpeted and non-carpeted surfaces were comparable. The success of this approach was evidenced by the observation that <300 µm dust loading rates did not change across 10 different categories of carpet cover, from <10% to >90% [22]. In contrast, for the purpose of the present study, use of the <80 µm fraction demonstrated more clearly the significance of carpets as a potential source of resuspended dust.

## 4. Conclusions

This study summarizes national trends in house dust concentrations and loadings of hundreds of organic and inorganic contaminants, many of which are harmful to human health (e.g., endocrine disruptors, neurotoxins, carcinogens, and asthma triggers). Spearman rank correlations yielded a number of testable hypotheses about indoor and outdoor sources of exposure in residential environments, some of which have not been identified previously.

1. Concentrations of many chemicals in house dust correlate negatively with construction date (i.e., increase with house age), while other contaminants correlate positively with construction date. Understanding how house age correlates with other building characteristics (such as %hardwood flooring versus %carpet) assists in interpreting these opposing trends.
  - Hardwood flooring, which is a manufactured wood product, correlates with increasing house age, and appears to be a source of metal(loid)s, phthalates, OPE flame retardants/plasticizers, and obsolete organochlorine pesticides such as ∑DDT (but not halogenated flame retardants). This finding suggests that other manufactured wood products (e.g., wood panelling, furniture) also act as indoor sources of contaminants in house dust.
  - In contrast, percent carpet cover decreases with house age (i.e., increases with construction date) and positively correlates with dust concentrations of BPA and BPS plasticizers, halogenated flame retardants, including BDEs, plus bromine and boron (but not ∑DDT or OPE flame retardants/plasticizers).
  - The observation of distinct trends in dust chemistry associated with carpeted floors (versus smooth floors) suggests that carpets themselves are a source of contaminants.
2. Different home heating styles correlate positively or negatively with proximity to the urban core (characterized by greater prevalence of older housing), and each heating style is associated with its own range of organic and inorganic contaminants in dust.
  - Usage of electricity and baseboards increases with proximity to the urban core, and is associated with increased dust concentrations of metals used in electrical applications (e.g., germanium and tin) and OPEs used in thermoplastics (e.g., TPHP).
  - In contrast, usage of natural gas and forced air heat distribution increases with proximity to the suburban fringe, and both are associated with chemicals used in heat-resistant alloys, rubber and plastics. Many of these chemicals are “emerging” and “alternative” flame retardants and plasticizers, but legacy flame retardants such as BDEs are also associated with these heating styles.
  - Determining whether contaminants originate from the fuel or from the heating system is a challenge. While there is evidence that fuel itself may contribute contaminants to house dust (mercury is used as an example), this study also presents evidence that components of heating appliances (including furnaces, stoves and fireplaces) and heat distribution systems (including forced air, radiators and baseboards) contribute heat-resistant chemicals and alloys to settled dust. In addition, forced air may re-distribute particle-bound substances from a variety of sources throughout the house.
3. Spearman rank correlations between contaminant concentrations and presence of children, pets and reported use of consumer products suggest a number of indoor sources, which point to future research directions:
  - As the number of children in the home increase, dust concentrations of numerous BDEs and OPEs increase, likely related to the use of flame retardants and plasticizers in children’s toys and other products.
  - While house cats are regarded as sentinels of exposure to OPEs, this study suggests that cat products actually introduce OPEs and numerous other potentially toxic organic compounds into the home.
  - A wide variety of synthetic organic compounds in dust are significantly associated with the use of room deodorizers/air fresheners.
4. Increased dust loading, which is associated with carpets, proximity to industry, infrequent cleaning and other factors, increases the risk of exposure to a complex mixture of contaminants in house dust.
  - For example, loadings of summed OPEs, BDEs and nonylphenols were significantly elevated in heavily carpeted children’s homes located near industry, an observation that has relevance to environmental equity studies and for effectively targeting exposure mitigation efforts.
  - Loading metrics emphasize the importance of carpets as a repository for dust contaminants that can become resuspended following activity in carpeted areas. While loading is a useful exposure predictor, concentration metrics are useful for identifying contaminant sources, as previously shown for metals.
  - Use of a finer dust fraction (<80 µm in this study) clarified the importance of carpets as a dust repository, as the finer fraction displayed a significant positive correlation with %carpet in CHDS homes, whereas the coarser faction (<300 µm) did not.
  - Depending on the chemical (and its source), increased dust mass loading may enrich or dilute chemical concentrations in dust.
5. More targeted study designs are needed to improve the characterisation of hidden indoor sources such as flame retardants used in building materials, or undisclosed ingredients used in air fresheners.
  - Numerous metal(loid)s are significantly correlated with flame-retardant compounds that appear to be used in building materials and heating systems.
  - Some substances that are enriched in house dust (due to indoor sources) displayed few to no correlations with the house characteristics captured by the CHDS questionnaire. Examples include triclosan, which is an antibacterial agent, and zinc, which has a wide range of indoor applications.
  - Detailed forensic approaches, which were outside the scope of this national baseline study, are needed to identify such specific indoor sources of exposure.

## Figures and Tables

**Table 1:** Characteristics of Canadian House Dust Study homes discussed in paper (either measured or captured by administering questionnaire during home visit). Numerical coding is indicated for yes/no and ranked answers.

Characteristic	Description
Construction Date	Year the house was built (i.e., higher value = newer home).
Municipal Zone	Location within the city; ranked 1 to 5 (1 = urban core to 5 = rural fringe).
#Children	Number of children in the dwelling.
#Dogs	Number of dogs in the dwelling.
#Cats	Number of cats in the dwelling.
Cigarette	Number of occupants that smoke cigarettes.
Cigar/Pipe	Number of occupants that smoke either a pipe or cigar.
Deodorizers	Are deodorizers/air fresheners used in the dwelling? (no = 0; yes = 1)
Upholstery treatments	Are upholstery treatments used in the dwelling? (no = 0; yes = 1)
Insecticide	Are insecticides used in the dwelling? (no = 0; yes = 1)
Candles	Are candles used in the dwelling? (no = 0; yes = 1)
%Vinyl Floor	Measured fraction of the sampled area covered in vinyl or linoleum flooring.
%Carpet	Measured fraction of the sampled area covered in carpet.
%Hardwood	Measured fraction of the sampled area covered in hardwood flooring.
Wood Heat	Does the house use wood as a fuel for heating? (no = 0; yes = 1)
Gas Heat	Does the house use gas as a fuel for heating? (no = 0; yes = 1)
Electric Heat	Does the house use electricity as a fuel for heating? (no = 0; yes = 1)
Oil Heat	Does the house use oil as a fuel for heating? (no = 0; yes = 1)
Forced Air	Is heat distributed by a forced air furnace? (no = 0; yes = 1)
Baseboard	Is heat distributed by electric baseboard heaters? (no = 0; yes = 1)
Radiator	Is heat distributed by hot water radiators? (no = 0; yes = 1)
Fireplace	Is a fireplace used for heat? (no = 0; yes = 1)
Stove	Is a stove used for heat? (no = 0; yes = 1)
Attached garage	Is garage attached to dwelling? (no = 0; yes = 1)
Proximity to industry	Is there industry of any type within 2 km of the dwelling? (no = 0; yes = 1)
Dust loading rate	Mass of dust per square meter per day (mg/m^2^/day).

**Table 2:** Significant correlations ^a^ between chemical concentrations in house dust and house characteristics, according to classes of chemicals determined in the Canadian House Dust Study (based on Spearman *rho* and *p* values listed in SI-1 and SI-2). Correlations may be positive or negative for construction date, municipal zone, and dust loading.

Chemical Class	Published Datasets ^d^	Characteristics Displaying Significant Correlations ^a^
**Larger CHDS Datasets (*n* = 642–1025 homes)**		
Polybrominated diphenyl ether (BDE) flame retardants (13 compounds; 642 homes)	[50]	#children; upholstery treatment; deodorizers; construction date; %carpet; gas heating; forced air
Non-BDE halogenated flame retardants (20 compounds; 642 homes)	[45]	#children; #cats; candles; upholstery treatment; deodorizers; construction date; municipal zone; attached garage; %carpet; gas heating; electric heating; forced air; baseboard; stove heating; radiators; dust loading
Organophosphate Esters (13 compounds; 816 homes)	[44]	#children; #cats; proximity to industry; candles; deodorizers; municipal zone; %hardwood; %vinyl; gas heating; dust loading
Pesticides (24 compounds; 913 homes)	[53]	#children; #cats; insecticides; construction date; municipal zone; %carpet; %hardwood; wood heating; gas heating; oil heating; forced air; radiators; dust loading
Bisphenol A, Octylphenol & Nonylphenols (6 compounds; 863 homes)	[51]	#cats; candles; deodorizers; %carpet; %vinyl; electric heating; radiators; municipal zone; dust loading
Metal(loid)s (59 elements; 1025 homes)	[17,22,47,55]	#children; #dogs; #cats; #cigarette smokers; proximity to industry; candles; deodorizers; insecticides; upholstery treatment; construction date; municipal zone; attached garage; %carpet; %hardwood; %vinyl; wood heating; gas heating; electric heating; oil heating; forced air; baseboard; fireplace; stove heating; radiators; dust loading
**Smaller CHDS datasets (*n* = 119–263 homes)**		
Phthalates (29 compounds; 128 homes)	[49,52]	construction date; municipal zone; dust loading
Musks (13 compounds; 198 homes)	[48]	candles; deodorizers; upholstery treatment; construction date; municipal zone; electric heating; baseboard
Parabens (and Triclosan ^c^) (7 compounds; 263 homes)	[43]	proximity to industry; construction date; fireplaces; dust loading
BPAA (BPA analogues) (15 compounds; 119 homes)	[46]	#cigar smokers; forced air ^b^; dust loading
Aryl and Alkyl-Aryl Phosphates (18 compounds; 143 homes)	[54]	deodorizers; construction date; wood heat; radiators; dust loading

^a^ Correlations were considered significant if two or more compounds in the class correlated at *p* < 0.05 or better with given characteristic; ^b^ BPAA was an exception: forced air correlated with only one compound of the BPAA class (BPM; r = 0.4; *p* < 0.01); ^c^ triclosan was determined but displayed no significant correlation with any house characteristic; ^d^ data summaries are provided in SI-6 to SI-16 for all substances.

**Table 3:** Relationships between selected characteristics of Canadian House Dust Study homes (*n* = 1025) identified using Spearman rank correlation analysis (** indicates *p* < 0.01; * indicates *p* < 0.05). No correlation was observed between construction date and proximity to industry; see SI-3 for all relationships between house characteristics.

Characteristic	Correlated Characteristic	rho (r)
Construction date	Attached garage	+0.44 **
	Municipal zone	+0.28 **
	Fireplace	+0.23 **
	%Carpet	+0.20 **
	%Hardwood	−0.25 **
	Radiators	−0.15 **
	Oil fuel	−0.13 **
	Wood fuel	−0.07 *
Municipal zone	Forced air	+0.18 **
	Gas fuel	+0.10 **
	Stove heat	+0.07 *
	Electric heat	−0.19 **
	Baseboards	−0.17 **
Candle use	Deodorizer use	+0.40 **
%Carpet	%Hardwood	−0.79 **
	%Vinyl	−0.09 **
Dust loading (80 µm)	Proximity to industry	+0.16 **
	%Carpet	+0.10 **
	Construction date	−0.19 **

**Table 4:** Selected contaminants associated with increasing house age and/or proximity to the urban core, and related building characteristics, identified using Spearman rank analysis ^a^. Negative correlations with construction date indicate that concentrations increase with increased house age; negative correlations with municipal zone indicate concentrations increase towards the urban core.

Class	Construction Date	Municipal Zone	%Hardwood	Oil Heat	Radiators	Wood Fuel	Electric Heat	Baseboard Heaters
**Organo-phosphate flame retardants/plasticizers**	--ODPP ** -EHDPP ** -TXP *	-EHDPP ** -TPHP ** -TBOEP ** -TCrP *	+TPHP ** +TnBP ** +TBOEP *	none	++BDPHP ** +24DIPPDPP & BIPPPP *	+TnBP * ++BtBPPP ** +BDPHP * +tBPDPP *	+TPHP *	+TPHP *
**Phthalates**	--DBP *	--DBP ** --BzBP * -DIBP * -DOP *	++DIBP **	none	+DCHP *	++DCHP *	none	none
**Musks**	---MX ** --MK **	--ADBI ** --MK ** --ATII ** -MX *	none	none	+MK **	none	+MK * +MX *	+MK * +MX *
**Pesticides**	---*p,p’*-DDD ** ---*o,p’*-DDT ** ---*p,p’*-DDE ** ---*p,p’*-DDT ** -*o,p’*-DDE ** -*o,p’*-DDD *	-*p,p’*-DDE ** -*o,p’*-DDT * -Parathion *	+*p,p’*-DDD ** +*o,p’*-DDT ** +*p,p’*-DDT ** +*p,p’*-DDE **	+Parathion ** +*o,p’*-DDT ** +*o,p’*-DDD * +Permethrin *	+*p,p*-DDE ** +*p,p*-DDT ** +Parathion ** +Aldrin * +*p,p’*-DDD * +*o,p’*-DDT * +*o,p’*-DDD *	+*o,p’*-DDT ** +*p,p’*-DDD **	+Chlorpyrifos *	none
**Metal(oid)s**	-----Pb **(r=-.53) ---Hg ** (r=-.38) --Co **; --Cd ** --Zn **; --Tl ** -Ge **; -Sr ** -Sn **; -Se ** -Ag **; -Ti ** -Mn **; -Al ** -REE **; -Cs ** -As * (and 17 others ^¥^)	---Ge ** --Sn **; --Hg ** -Co **; -Ag ** -Pb **; -Sr ** -S **; -Se ** -Na ** -Cu *	++Sr **; ++Pb ** ++Co **; ++Ti ** ++Sn **; ++Ge ** ++Y **; ++Tl ** ++REE ** +Zn **; +Rb **; +Cu **; +Mn ** (and 11 others ^¥^)	+Cd **; +Ge ** +S **; +Cs **, +Rb **; +Li ** +REE ** +Mn *; +Zn * +Hg *	+Pb ** +Co *; +Hg * +Fe *; +Ag *	+Cs **; +S ** +Hg **; +Y ** +Ag **; +Ga ** +Mn ** +REE ** +Ge *; +Cd * +Ta *	+Ge **; +Sn ** +Sr **; +As ** +Cu **; +Rb ** +REE ** +Al *; +S *; +Ti * +Y *; +Ag * +Sb *	++Ge ** +Sn ** +REE ** +Ag *;+As * +Sb *,+S * +Cu *

^a^ Spearman rank results; ** indicates *p* < 0.01; * indicates *p* < 0.05; Single “+” means positive *rho* < 0.20; “++” *rho* from 0.20 to 0.29; Single “-” means negative *rho* < −0.20; “--” *rho* from −0.20 to −0.29, “---” *rho* from −0.30 to −0.39; “-----” *rho* from −0.50 to −0.59; ^¥^ See SI-1 and SI-2 for all Spearman rank correlations.

**Table 5:** Selected contaminants associated with increasing construction date and/or proximity to suburban fringe, and related building characteristics, identified using Spearman rank analysis ^a^. Positive correlations with construction date indicate that concentrations increase in newer homes; positive correlations with municipal zone mean concentrations increase towards suburban fringe. See SI-01 to SI-02 for all Spearman rank correlations.

Class	Construction Date	Municipal Zone	Attached Garage	%Carpet	Fireplace	Forced Air	Gas Fuel	Stove Heat
**Halogenated Flame Retardants**	+BDE-17 ** +BDE-71 ** +TBCT ** +ATE **	+ATE **	+BDE-17 ** +TBCT * +HBB *	+BDE-17 ** +BDE-28 ** +(α+β)-TBECH ** +α-TBECH ** +BATE ** +β-TBECH ** +(*syn+anti*)-DP ** +BTBPE ** +*anti*-DP ** +*syn*-DP *; +ATE * +BDE-99 * +BDE-47 *	none	+BDE-209 * +BDE-99 * +TBCT ** +(ɣ + δ)-TBECH ** +β-TBECH ** +α-TBECH ** +BTBPE ** +BATE ** +HBB *	+BDE-100 * +BDE-28 * +BDE-99 * ++(ɣ+δ)-TBECH ** +α-TBECH ** +β-TBECH ** +*syn*-DP ** +(*syn+anti*)-DP ** +*anti*-DP **	+PBT ** +EHTBB * +HBB *
**Organo- phosphate flame retardants/plasticizers**	++IDDPHP *	none	none	none	++IDDPHP *	none	++TCEP ** +TCPP ** +TiBP ** +EHDPP *	++BDPHP **
**BPA and BPA analogues**	+BPS * +BPA *	-BPA *	none	++BPS ** +BPA **	+BPS *	++++BPM **	none	none
**Octylphenol & Nonylphenols**	none	none	none	+NP2EO **	none	++4-*tert*-OP **	+NP2EO **	none
**Metal(loid)s**	++B ** +Br **; +Hf **	+B **; +Br ** +Hf **; +Mo ** +Ta **; +Mg * +Mn *; +U *	++B ** +Au **; +Br ** +Hf **; +Mg ** +Mo **; +Te **	++B **; ++Br ** +Na **; +Bi ** +Mo *	+B ** +Ta ** +Br * +Mo *	+++Hf **; +++Te ** +++Rb ** ++Mg **; ++Mo ** ++Mn **; ++REE ** +Br **	++B **; ++Mo ** +Br **; +Mg ** +Ta **	++Mn ** +Rb **

^a^ Spearman rank results; ** indicates *p* < 0.01; * indicates *p* < 0.05; Single “+” means positive *rho* < 0.20; “++” *rho* from 0.20 to 0.29; “+++” *rho* from 0.30 to 0.39; “++++” *rho* from 0.40 to 0.49; same range for “-” *rho* value.

**Table 6:** Metal(loid)s associated with organic compounds (*p* < 0.05) that correlate with common flooring or heating characteristics in CHDS homes. Superscripts indicate which characteristics are significantly associated (*p* < 0.05) with that organic compound: 1 = %hardwood, 2 = %vinyl, 3 = forced air + gas heating, 4 = electric + baseboard, 5 = radiator, 6 = stove, 7 = wood.

Polybrominated Diphenyl Ether (BDE) Flame Retardants	Non-BDE Halogenated Flame Retardants	Organophosphate Esters (OPEs)
**^3^BDE-99:** As; Bi; Br; Cr; Ni **^3^BDE-28:** Bi; Br; K; Sb **^3^BDE-100:** As; Bi; Br; Sb **^3^BDE-209:** Br; Cr; Cu; Hf; Mo; Ni; Pb; Sb; Sn; Zn **^4^BDE-71:** Bi; Br; Sb; Sc	**^2^PBB:** As; Br; Cd; Cu; Fe; Hf; Pb; Sb; Sn; Zn ^**3,5**^**TBECH isomers:** Ba; Bi; Br; Cr; Hf; Mo; Ni; Re; Sc; Se; Te **^3^Dechlorane Plus (DP) isomers:** Ag; Bi; Br; Cd; Co; Cr; Cu; Fe; Mo; Ni; Pb; Re; Sb; Zn **^3^TBCT:** Al; Be; Bi; Br; Cr; Cu; Eu; Fe; Hf; K; Na; Rb; Sb; Sc **^3^BTBPE:** Br; Cd; Cr; Cu; Hg; Ni; Sb; Zn **^3^BATE:** Be; Br; Bi; Hf; K; Na; Re **^3^HBB:** As; Br; Cr; Eu; Fe; Sb; Sc **^4,6^PBT:** Cs; Ge; Mn; Sn **^4^TBCT:** Al; Be; Bi; Br; Cr; Cu; Eu; Fe; Hf; K; Na; Rb; Sb; Sc **^4,6^HBB:** As; Br; Cr; Eu; Fe; Sb; Sc **^5^TBpX:** Br; Cd; Ce; La; Mo; Nd **^5^PBEB:** As; Cd; Cr; Hg; Zn **^6^EHTBB:** As; Bi; Cs; Ni; Re	**^1,2^TBOEP** Ag; Ba; Br; Co; Cu; Ge; Hf; K; Ni; Re; Sb; Sn; Zn **^1,2,7^TnBp:** Al; Ba; Cs; Ge; Li; Sn; Ta; Ti; Tl; Th; U; Y and REE (La; Ce; Pr; Er; Tb; Dy; Ho; Sm; Yb) **^1,4^TPHP:** Ag; Au; Bi; Cd; Cr; Cu; Ge; Hg; Ni; Pb; Re; Sb; Sn; Ti; Zn **^3^TCEP:** Au; Ba; Bi; Br; Mo; Ta; U **^3^TCPP:** Au; Bi; Br **^3^TiBP:** Cs; Li; Mg; Mo; P; Ta; U **^3^EHDPP:** Ag; Au; Ba; Cd; Cr; Hg; Mo; Pb; Zn

**Table 7:** Relationships between contaminant concentrations in dust and characteristics related to consumer products identified using Spearman rank correlation analysis.

Characteristic	Halogenated Flame Retardants	Organophosphate Flame Retardants/Plasticizers	Bisphenol A, Octylphenol & Nonylphenols	Pesticides	Metals and Metalloids	Musks
**# Children ^a^**	+BDE-99 **; +BDE-100 ** +BDE-85 **; +BDE-47 ** +BDE-17 **; +BDE-71 ** +BDE-154 *; +BDE-28 * +BDE-153 *; +BDE-66 *	++TBOEP ** +TDCPP ** +TiBP *	+4-*tert*-OP *	+HCB ** +*o,p’*-DDD ** +*o,p’*-DDE *	+B **; +Be **; +Ca **; +Hf ** +K **; +Mg **; +Nb **; +Rb ** +Sr **; +Te **; +Tl **; +Y **; +Ba *; +Ga *; +Mn * +U **; +Th *	none
**Upholstery Treatment ^a,b^**	+BDE-28 **; +BDE-85 ** +BDE-209 *; +BDE-17 ** +BDE-47 *; +BDE-99 * +BDE-100 *; +BDE-153 * +BDE-154 *; +BTBPE ** +α-TBECH ** +(α+β)-TBECH *	none	+4-*tert*-OP *	none	+B **; +Br **; +Mo **; +Se **; +Ni **; +Te **; +Bi *; +Co *; +Fe *; +Hf *	+OTNE ** +HHCB **
**# Cats**	+α-TBECH ** +(α+β)-TBECH * +β-TBECH *	++TnBP ** +TCEP ** +TCPP ** +TEP **	+NP1EO ** +NP2EO *	+*o,p’*-DDT ** +*p,p’*-DDD ** +*p,p’*-DDT * +*o,p’*-DDD *	++++U **; +++Th **; ++Al ** +Ba **; +Cs **; +Fe **; +Ga ** +K **; +Li **; +Nb **; +P ** +Rb **; +Sr **; +Ta **; +Tl ** +Y **; +Mn *; +Be *	+MT *
**Deodorizers ^c^**	++TBCT ** +BDE-71 ** +BDE-66 * +(*syn+anti*)-DP * +*syn*-DP * +EHTBB * +*anti*-DP *	+EHDPP ** +TCPP ** +TBOEP ** +TCEP ** +TPHP ** +TDCPP * +TEP * + TIPPP * + RBDP * +IDDPHP *	++++NP2EO ** +++NP1EO ** +++BPA ** ++Branched-NP ** ++4-*tert*-OP ** +*n*-NP **	+Malathion **	++Bi **; ++B **; +Al **; +Co **; +Cu ** +Na **; +Re **; +Sb ** +K *;+Se *	+++OTNE ** ++HHCB-lactone ** ++HHCB ** ++ATII ** +AHTN **
**Candles ^c^**	+TBCT ** +EHTBB *	+TBOEP ** +TPHP **	++BPA ** ++NP2EO ** ++NP1EO ** ++Branched-NP ** ++4-*tert*-OP ** +*n*-NP **	+*p,p’*-DDT **	++Na ** +Al **;+Bi **; +Ge **; +Re ** +Ag *; +Co *	+ADBI ** +AHTN ** +HHCB * +MK * +HHCB-lactone * +OTNE *
**Insecticide** **Use ^b^**	none	none	none	+Diazinon ** +HCB ** +*o,p’*-DDT ** +*p,p’*-DDD * +Permethrin *	++Te ** +Ca **; +Mg **; +Mn ** +Mo **; +Sr **; +Tl **; +Y ** +Hf *; +Rb *	none

(** indicates *p* < 0.01; * indicates *p* < 0.05). Single “+” means positive *rho* < 0.20; “++” *rho* from 0.20 to 0.29; “+++” *rho* from 0.30 to 0.39; “++++” *rho* from 0.40 to 0.49. ^a^ Number of children correlates positively with upholstery treatments (*p* < 0.05) and construction date (*p* < 0.05); ^b^ municipal zone correlates positively with use of upholstery treatments (*p* < 0.05) and insecticides (*p* < 0.01); ^c^ municipal zone correlates negatively with use of candles (*p* < 0.05) and deodorizers (*p* < 0.05).

**Table 8:** Comparison of metrics (loading versus concentration) for determining influence of house characteristics on combined exposures to organic compounds in children’s homes shown in Figure 2. (*n* = number of homes in each subset; 80 µm dust size fraction).

Metric	Median Daily Loading (ng/m^2^/day)			Median Concentration (mg/kg)		
House Characteristics	Away (>2 km) from Industry; 0–18% Carpet	Close (<2 km) to Industry; 75–100% Carpet	Mann–Whitney U-Test for Significance (*p* Value)	Away (>2 km) from Industry; 0–18% Carpet	Close (<2 km) to Industry; 75–100% Carpet	Mann–Whitney U-Test for Significance (*p* Value)
**BDE flame retardants ^a^**	5.57	24.5	*p* = 0.014	1.714	1.898	*p* = 0.226
	(*n* = 32)	(*n* = 18)		(*n* = 32)	(*n* = 18)	
**Non-BDE** **halogenated flame retardants ^b^**	1.16	3.24	*p* = 0.106	0.299	0.367	*p* = 0.571
	(*n* = 32)	(*n* = 18)		(*n* = 32)	(*n* = 18)	
**Organophosphate esters ^c^**	360	760	*p* = 0.002	74.0	75.3	*p* = 0.928
	(*n* = 41)	(*n* = 26)		(*n* = 41)	(*n* = 26)	
**Nonylphenols ^d^**	70	310	*p* < 0.001	21.6	22.4	*p* = 0.353
	(*n* = 47)	(*n* = 25)		(*n* = 47)	(*n* = 25)	

^a^ sum of BDE-100; BDE-138; BDE-153; BDE-154; BDE-17; BDE-183; BDE-209; BDE-28; BDE-47; BDE-66; BDE-71; BDE-85; and BDE-99; ^b^ sum of α-TBECH; β-TBECH; TBpX; syn-DP; TBCT; (α + β)-TBECH; (ɣ + δ)-TBECH; (syn + anti)-DP; anti-DP; BTBPE; DPTE; EHTBB; HBB;PBB;PBBA;PBBB; PBEB;PBT;BATE; and ATE; ^c^ sum of EHDPP; TBOEP; TCEP; TCPP; TCrP; TDCPP; TEP; TiBP; TMP; TnBP; TPeP; TPHP; and TPrP; ^d^ sum of Branched-NP; *n*-NP; NP1EO; and NP2EO concentrations in each home.

## Data Availability

Research data are not shared.

## Supplementary Materials

The following supporting information can be downloaded at: https://www.mdpi.com/article/10.3390/ijerph191610329/s1, SI-1a: Large datasets: Spearman rank correlations between dust concentrations and heating fuel and heat distribution style; SI-1b: Large datasets: Spearman rank correlations between dust concentrations and construction date, flooring and other characteristics; SI-1c: Large datasets: Spearman rank correlations between dust concentrations and proximity to industry, inhabitants, and smoking behaviour; SI-1d: Large datasets: Spearman rank correlations between dust concentrations and consumer products and dust mass loading rate; SI-2a: Small datasets: Spearman rank correlations between dust concentrations and heating fuel and heat distribution style; SI-2b: Small datasets: Spearman rank correlations between dust concentrations and construction date, flooring and other characteristics; SI-2c: Small datasets: Spearman rank correlations between dust concentrations and proximity to industry, inhabitants, and smoking behaviour; SI-2d: Small datasets: Spearman rank correlations between concentrations and consumer products and dust loading rate; SI-3: Relationships between house characteristics; SI-4: Heating styles documented for CHDS homes, 2007-2010; SI-5: Influence of carpets on dust loading rates (mg/m^2^/day) in Canadian House Dust Study; SI-6: Concentrations and loadings (<80 µm fraction) for BDE Flame retardants determined in subset of 642 Canadian House Dust Study samples; SI-7: Concentrations and loadings (<80 µm fraction) for non-BDE halogenated flame retardants determined in 642 Canadian House Dust Study samples; SI-8: Concentrations and loadings (<80 µm fraction) for organophosphate esters determined in subset of 816 Canadian House Dust Study samples; SI-9: Concentrations and loadings (<80 µm fraction) for pesticide compounds determined in Canadian House Dust Study samples from 913 homes; SI-10: Concentrations and loadings (<80 µm fraction) for BPA, nonylphenols and octylphenol determined in 863 Canadian House Dust Study samples; SI-11: Concentrations and loadings (<80 µm fraction) for phthalates determined in 128 Canadian House Dust Study samples; SI-12: Concentrations and loadings (<80 µm fraction) for synthetic musks determined in 198 Canadian House Dust Study samples; SI-13: Concentrations and loadings (<80 µm fraction) for parabens and triclosan determined in 263 Canadian House Dust Study samples; SI-14: Concentrations and loadings (<80 µm fraction) for BPA analogues (BPAA) determined in 119 Canadian House Dust Study samples; SI-15: Concentrations and loadings (<80 µm fraction) for aryl and alkyl-aryl phosphates determined in 143 Canadian House Dust Study samples; SI-16: Total concentrations (<80 µm fraction) for metals and metalloids determined in Canadian House Dust Study samples. SI-17: Chemicals and building materials lacking associations.
