# Supplementary material for: Relationships between House Characteristics and Exposures to Metal(loid)s and Synthetic Organic Contaminants Evaluated Using Settled Indoor Dust
Source: Int J Environ Res Public Health. 2022 Aug 19;19(16):10329. doi: 10.3390/ijerph191610329 (PMC9408639; doi:10.3390/ijerph191610329)
Supplement: Supplementary file 1 [file ijerph-19-10329-s001.zip › ijerph-1819865-supplementary.pdf]

Online supporting information for the following article published in *IJERPH* (2022):

Relationships between House Characteristics and Exposures to Metal(loid)s and Synthetic Organic Contaminants Evaluated Using Settled Indoor Dust

Pat E. Rasmussen [1,2], Cariton Kubwabo [1], H. David Gardner [1,2], Christine Levesque [1] and Suzanne Beauchemin [1,*]

[1] Environmental Health Science and Research Bureau, HECS Branch, Health Canada, Ottawa, ON K1A 0K9, Canada
[2] Department of Earth and Environmental Sciences, University of Ottawa, Ottawa, ON K1N 9A7, Canada
* Correspondence: suzanne.beauchemin@hc-sc.gc.ca

List of Supplementary Information

SI-1a Large datasets: Spearman rank correlations between dust concentrations and heating fuel and heat distribution style
SI-1b Large datasets: Spearman rank correlations between dust concentrations and construction date, flooring and other characteristics;
SI-1c Large datasets: Spearman rank correlations between dust concentrations and proximity to industry, inhabitants, and smoking behavior;
SI-1d Large datasets: Spearman rank correlations between dust concentrations and consumer products and dust mass loading rate;
SI-2a Small datasets: Spearman rank correlations between dust concentrations and heating fuel and heat distribution style;
SI-2b Small datasets: Spearman rank correlations between dust concentrations and construction date, flooring and other characteristics;
SI-2c Small datasets: Spearman rank correlations between dust concentrations and proximity to industry, inhabitants, and smoking behavior;
SI-2d Small datasets: Spearman rank correlations between concentrations and consumer products and dust loading rate;
SI-3 Relationships between house characteristics;
SI-4 Heating styles documented for CHDS homes, 2007-2010;
SI-5 Influence of carpets on dust loading rates (mg/m^2/day) in Canadian House Dust Study;
SI-6 Concentrations and loadings (<80 µm fraction) for BDE Flame retardants determined in subset of 642 Canadian House Dust Study samples;
SI-7 Concentrations and loadings (<80 µm fraction) for non-BDE halogenated flame retardants determined in 642 Canadian House Dust Study samples;
SI-8 Concentrations and loadings (<80 µm fraction) for organophosphate esters determined in subset of 816 Canadian House Dust Study samples;
SI-9 Concentrations and loadings (<80 µm fraction) for pesticide compounds determined in Canadian House Dust Study samples from 913 homes;
SI-10 Concentrations and loadings (<80 µm fraction) for BPA, nonylphenols and octylphenol determined in 863 Canadian House Dust Study samples;
SI-11 Concentrations and loadings (<80 µm fraction) for phthalates determined in 128 Canadian House Dust Study samples;
SI-12 Concentrations and loadings (<80 µm fraction) for synthetic musks determined in 198 Canadian House Dust Study samples;
SI-13 Concentrations and loadings (<80 µm fraction) for parabens and triclosan determined in 263 Canadian House Dust Study samples;
SI-14 Concentrations and loadings (<80 µm fraction) for BPA analogues (BPAA) determined in 119 Canadian House Dust Study samples;
SI-15 Concentrations and loadings (<80 µm fraction) for aryl and alkyl-aryl phosphates determined in 143 Canadian House Dust Study samples;
SI-16 Total concentrations (<80 µm fraction) for metals and metalloids determined in Canadian House Dust Study samples.
SI-17 Chemicals and building materials lacking associations.

SI-1a Large datasets: Spearman rank correlations[a] between dust concentrations and heating fuel and heat distribution style (*indicates significant at $p < .05$; ** indicates significant at $p < .01$).

	**Polyrominated diphenyl ethers (BDE) Flame retardants**	**Non-BDE Halogenated Flame retardants**	**Organophosphate esters (OPE flame retardants and plasticizers)**	**Pesticides**	**Bisphenol A, Octylphenol & Nonylphenols**	**Metal(loid)s (45 elements excluding lanthanides)**	**Lanthanides (14 elements)**
**Heating Fuel**							
**wood**	0 compounds	0 compounds	+TnBP*	+*o,p′*-DDT**<br>+*p,p′*-DDD**	0 compounds	+Cs**; +S**; +Hg**; +Y**; +Ag**; Ga**; +Mn**<br><br>+Cd*; +Ta*; +Ge*	+Yb**; +Tb**; +Er**; +Dy**; +Sm**; +Gd**; +Eu**; +Ho**
**gas**	+BDE-99*<br>+BDE-28*<br>+BDE-100*	++(γ+δ)-TBECH**<br>+α-TBECH**<br>+β-TBECH**<br>+*syn*-DP**<br>+(*syn+anti*)-DP**<br>+*anti*-DP**	++TCEP**<br>+TCPP**<br>+TiBP**<br>+EHDPP*	+HCB**<br>+Malathion**<br>+Triallate**<br>+*o,p′*-DDE*	+NP2EO**	++B**; ++Mo**; +Mg**; +Ba**; +Ta**; +Re**; +Br**; +Cr**	0 compounds
**electric**	+BDE-71**	+TBCT**<br>+PBT*<br>+HBB*	+TPHP*	+Chlorpyrifos*	+BPA**<br>+*n*-NP*	+Ge**; Sn**; +As**; +Sr**; +Cu**; +Rb**<br><br>+Ti*; +Al*; +S*; +Y*; +Ag*; +Sb*	+Eu**; +Sm**; +Dy**; +Nd**<br><br>+Yb*; +Pr*; +Er*; +Gd*
**oil**	0 compounds	0 compounds	0 compounds	+Parathion**<br>+*o,p′*-DDT**<br>+Permethrin*<br>+*o,p′*-DDD*	0 compounds	+Ge**; +S**; +Cs**; +Cd**; +Rb**; +Li**<br><br>+Hg*; +Mn*; +Zn*	+Nd**<br><br>+Gd*; +Yb; +Th*; +Dy* +Ho*; +Er*; +Sm*; +Eu*
**Heat Distribution Style**							
**forced air**	+BDE-209*<br>+BDE-99*	+(γ+δ)-TBECH**<br>+β-TBECH**<br>+α-TBECH**<br>+TBCT**<br>+BTBPE**<br>+BATE**<br>+HBB*	0 compounds	+HCB**;+Aldrin**<br>+*cis*-Chlordane**<br>+Heptachlor**<br>+*p,p′*-DDT**<br>+Heptachlor Epoxide**<br>+Terbufos**;<br>+*o,p′*-DDE**<br>+Triallate**;<br>+Malathion**<br>+Dieldrin*<br>+*cis*-Nonachlor*	++4-*tert*-OP**	+++Hf**; +++Te**; +++Rb**;<br><br>++Mg**; ++Mo**; ++Mn**; ++Y**; ++Be**; ++U**<br><br>+Br**; +K**; +As**; +Fe**; +Sc**; +Tl**; +Nb**; +Cr**; +Ca**; +Ba**; +Ni**; +Co**; +Cd**; +Th**<br><br>+Na*	++Nd**; ++Ho**; ++Sm**; ++Eu**; ++Er**; ++Gd**; ++Dy**; ++Pr**; ++Ce**<br><br>+Tm**; +Tb**; +La**
**baseboard**	+BDE-71*	+TBCT**<br>+PBT**	+TPHP*	0 compounds	0 compounds	++Ge**;+Sn**<br>+Ag*;+As*,+Sb*,+S*,+Cu*	+Eu**;+Sm*
**fireplace**	0 compounds	0 compounds	0 compounds	+Azinphos Methyl**	0 compounds	+Au**; +B**;+Ta**,<br>+Br*;+Mo*	0 compounds
**stove**	0 compounds	+PBT**<br>+EHTBB*<br>+HBB*	0 compounds	+*cis*-Nonachlor*	0 compounds	++Mn**<br>+Rb**; +Cs**; +Ge**; +As**; +K**; +Li*	0 compounds
**radiator**	0 compounds	+TBpX**<br>+PBEB*<br>+(α+β)-TBECH*<br>+α-TBECH*<br>+β-TBECH*	0 compounds	+*p,p′*-DDE**<br>+*p,p′*-DDT**<br>+Parathion**<br>+Aldrin*; +*p,p′*-DDD*<br>+*o,p′*-DDT*<br>+*o,p′*-DDD*	+NP2EO**<br>+branched-NP **<br>+4-*tert*-OP**<br>+NP1EO**<br>+*n*-NP*	+Pb**<br><br>+Co*; +Hg*; +Fe*; +Ag*	0 compounds

[a] Single "+" means positive $r_s < .20$; "++" means r_s from 0.20 to 0.29; "+++" means r_s from 0.30 to 0.39.

SI-1b Large datasets: Spearman rank correlations[a] between dust concentrations and construction date, flooring and other characteristics.
(*indicates significant at _p_ < .05; ** indicates significant at _p_ < .01).

	**Polybrominated diphenyl ethers (BDE) Flame retardants**	**Non-BDE Halogenated Flame retardants**	**Organophosphate esters (OPE flame retardants and plasticizers)**	**Pesticides**	**Bisphenol A, Octylphenol & Nonylphenols**	**Metal(loid)s including lanthanides (REE = rare earth elements, see Rasmussen et al., 2017 for details)**
**Construction date**	+BDE-71**<br>+BDE-17**	+TBCT**<br>+ATE**	-EHDPP**	---_p,p'_-DDD**<br>---_o,p'_-DDT**<br>---_p,p'_-DDE**<br>---_p,p'_-DDT**<br>-_o,p'_-DDE**<br>-Dieldrin**<br>-Chlorpyrifos**<br>-_cis_-Nonachlor**<br>-_cis_-Chlordane*<br>-_trans_-Nonachlor*<br>-_o,p'_-DDD*	+BPA*	++B**; +Br**; +Hf**<br><br>-----Pb**(r=-.53); ---Hg**(r=-.38)<br><br>--Co**; --Cd**; --Zn**; --Tl**<br><br>-Fe**; -V**; -Sr**; -Ba**; -P**; -Be**; -Mg**; -Sn**;<br>-Cs**; -Ga**; -Ag**; -Ge**; -Ti**; -Mn**; -Al**; -Li**;<br>-Re**; -Te**; -Se**; -REE**; -U**; -Th**<br><br>-Y*,-As*,-Ta*,-Nb*; -Mo*; -Ca*
**Municipal zone**	0 compounds	+ATE**<br>-PBT*	-TPHP**<br>-TBOEP**<br>-EHDPP**<br>-TCrP*	-_p,p'_-DDE**<br>-_o,p'_-DDT*<br>-Parathion*<br><br>+Heptachlor*	-Branched-NP**<br>-_n_-NP**<br>-NP1EO**<br>-BPA*<br>-NP2EO*	+B**; +Br**; +Hf**; +Mo**; Ta**; +Mg*; +Mn*; +U*<br><br>---Ge**<br>--Sn**; --Hg**<br>-Pb**; -Ag**; -S**; -Se**; -Sr**; -Na**; -Co**<br>-Cu*
**Attached garage**	+BDE-17**	+HBB*<br>+TBCT*	0 compounds	0 compounds	0 compounds	++B**<br><br>+Br**; +Te**; +Hf**; +Mg**; +Mo**; +Au**
**% Carpet**	+BDE-17**<br>+BDE-28**<br>+BDE-99*<br>+BDE-47*	+(α+β)-TBECH**<br>+α-TBECH**;<br>+BATE**<br>+β-TBECH**<br>+(_syn+anti_)-DP**<br>+BTBPE**<br>+_anti_-DP**<br>+_syn_-DP*<br>+ATE*	0 compounds	+Terbufos*<br>+Diazinon*	+NP2EO**<br>+BPA**	++B**; ++Br**<br><br>+Na**; +Bi**<br><br>+Mo*
**% Hardwood**	0 compounds	0 compounds	+TPHP**<br>+TnBP**<br>+TBOEP*	+_p,p'_-DDD**<br>+_o,p'_-DDT**<br>+_p,p'_-DDT**<br>+_p,p'_-DDE**	0 compounds	++Sr**; ++Pb**; ++Co**; ++Ti**; ++Sn**; ++Ge**;<br>++Y**; ++Tl**; ++REE**<br><br>+Ca**; +Fe**; +Ga**; +Be**; +Rb**; +V**; +Ba**;<br>+Cs**; +Li**; +Nb**; +Cu**; +Mn**; +Zn**; +Mg**<br><br>+Ta*
**% Vinyl**	0 compounds	+PBB*	+TBOEP*<br>+TnBP*	0 compounds	+Branched-NP**<br>+NP2EO**<br>+NP1EO**<br>+BPA**	+Hf*; +Mo*

[a] Single "+" means positive r_s < .20; "++" means r_s from 0.20 to 0.29; "+++" means r_s from 0.30 to 0.39; "++++" means r_s from 0.40 to 0.49;
(same ranges for "-" and "--", etc. negative r_s).

SI-1c Large datasets: Spearman rank correlations[a] between dust concentrations and proximity to industry, inhabitants, and smoking behaviour (*indicates significant at $p < .05$; ** indicates significant at $p < .01$).

	**Polybrominated diphenyl ethers (BDE) Flame retardants**	**Non-BDE Halogenated Flame retardants**	**Organophosphate esters (OPE flame retardants and plasticizers)**	**Pesticides**	**Bisphenol A, Octylphenol & Nonylphenols**	**Metal(loid)s excluding lanthanides (see Rasmussen et al. 2017 for lanthanides)**
**# Children**	+BDE-99**<br>+BDE-100**<br>+BDE-85**<br>+BDE-47**<br>+BDE-17**<br>+BDE-71**<br>+BDE-154*<br>+BDE-28*<br>+BDE-153*<br>+BDE-66*	+β-TBECH**<br>+EHTBB**<br>+HBB**<br>+(α+β)-TBECH**<br>+ATE**<br>+TBCT**<br>+α-TBECH*	++TBOEP**<br>+TDCPP**<br>+TiBP*	+HCB**<br>+*o,p′*-DDD**	+4-*tert*-OP*	+Y**; +K**; +Sr**; +Mg**; +Ca**; +Te**; +Rb**; +Tl**; +Be**; +Nb**; +B**<br><br>+U*; +Hf*; +Ga*; +Ba*;+Th*; +Mn*
**# Dogs**	0 compounds	0 compounds	0 compounds	0 compounds	+++*n*-NP**	+++K**<br><br>++Rb**; ++Tl**; ++Y**; ++V**; ++Al**; ++Be**; ++P**; ++Li**; ++Mn**<br><br>+Cs**; +Fe**; +Th**; +Ga**; +U**; +Nb**; +Mg**; +Ba**; +Sr**; +Ta**; +Na**; +Co**; +Hf**
**# Cats**	0 compounds	+α-TBECH**<br>+(α+β)-TBECH*<br>+β-TBECH*	++TnBP**<br>+TCEP**<br>+TCPP**<br>+TEP**	+*p,p′*-DDD**<br>+*o,p′*-DDT**<br>+*p,p′*-DDT**<br>+*o,p′*-DDD*	+NP1EO**<br>+NP2EO*	++++U**; +++Th**<br><br>++Al**<br><br>+Be**; +Ga**; +Li**; +Tl**; +P**; +Cs**; +Nb**; +Ta**; +K**; +Rb**; +Ba**; +Y**; +Sr**; +Fe**; +Mn*
**# Cigarette**	0 compounds	0 compounds	0 compounds	+Chlorpyrifos**	0 compounds	+K**; +P**; +Tl**; +Mo**; +Mg**; +Li**; +Rb**; +Mn**; +Nb**; +Cs**<br><br>+Fe*; +Be*; +Ba*; +Sr*;+U*
**# Cigar**	0 compounds	0 compounds	0 compounds	+*cis*-Chlordane*	0 compounds	0 compounds
**Within 2 km of any industry**	0 compounds	0 compounds	+TCEP**<br>+TCrP**<br>+TiBP*	0 compounds	0 compounds	+Nb**; +Cs**; +Li**; +Te**; +P**; +Re**; +Tl**; +Mo**; +U**; +Th**<br><br>+Mn*; +Ta*; +Mg*; +Ba*

[a] Single "+" means positive $r_s < .20$; "++" means r_s from 0.20 to 0.29; "+++" means r_s from 0.30 to 0.39; "++++" means r_s from 0.40 to 0.49.

SI-1d Large datasets: Spearman rank correlations[a] between dust concentrations and consumer products and dust mass loading rate (*indicates significant at $p < .05$; ** indicates significant at $p < .01$).

	Polybrominated diphenyl ethers (BDE) Flame retardants	Non-BDE Halogenated Flame retardants	Organophosphate esters (OPE flame retardants and plasticizers)	Pesticides	Bisphenol A, Octylphenol & Nonylphenols	Metal(loid)s excluding lanthanides (see Rasmussen et al., 2017 for lanthanides)
**Candle**	0 compounds	+TBCT**<br>+EHTBB*	+TBOEP**<br>+TPHP**	+*p,p'*-DDT**	++NP2EO**<br>++BPA**<br>++NP1EO**<br>++Branched-NP**<br>++4-*tert*-OP**<br>+*n*-NP**	++Na**<br><br>+Bi**; +Re**; +Ge**; +Al**; +Co*; +Ag*
**Deodorizer**	+BDE-71**<br>+BDE-66*	++TBCT**<br>+(*syn+anti*)-DP*<br>+*syn*-DP*<br>+EHTBB*<br>+*anti*-DP*	+TCPP**<br>+TBOEP**<br>+TCEP**<br>+TPHP **<br>+EHDPP**<br>+TDCPP*<br>+TEP*	+Malathion**	++++NP2EO**<br>+++NP1EO**<br>+++BPA**<br>++Branched-NP**<br>++4-*tert*-OP**<br>+*n*-NP**	++Bi**; ++B**<br><br>+Al**; +Cu**; +Re**; +Na**; +Co**; +Sb**<br><br>+K*,+Se*
**Insecticide**	0 compounds	0 compounds	0 compounds	+Diazinon**<br>+HCB**<br>+*o,p'*-DDT**<br>+Permethrin*<br>+*p,p'*-DDD*	0 compounds	++Te**<br><br>+Mg**; +Y**; +Sr**; +Ca**; +Tl**; +Mn**; +Mo**<br><br>+Rb*; +Hf*
**Upholstery**	+BDE-28**<br>+BDE-85**<br>+BDE-209*<br>+BDE-17**<br>+BDE-47*<br>+BDE-99*<br>+BDE-100*<br>+BDE-153*<br>+BDE-154*	+BTBPE**<br>+α-TBECH**<br>+(α+β)-TBECH*	0 compounds	0 compounds	+4-*tert*-OP*	+B**; +Br**; +Mo**; +Se**; +Ni**; +Te**<br><br>+Bi*; +Co*; +Hf*; +Fe*
**Dust loading rate**	0 compounds	+BATE**<br>+*syn*-DP*	-TPHP **<br>-TDCPP**<br>-TCrP**<br>-TCEP**<br>-TnBP**	+*p,p'*-DDT**<br>+*p,p'*-DDE**<br>+*p,p'*-DDD**<br>+*cis*-Nonachlor**<br>+*o,p'*-DDT*<br>+*o,p'*-DDE*<br><br>-Azinphos Methyl*	+4-*tert*-OP**<br>+*n*-NP**<br>+NP2EO**	+++K**; +++Cs**<br><br>++Li**; ++Tl**; ++Rb**; ++Be**; ++U**; ++Th**; ++Al**;<br><br>+Mn**; +Ca**; +P**; +Mg**; +Fe**; +Na**; +Co**; +Nb**; +Se**; +V**; +Y**; +As**; +Ta**; +Ba**; +Ga**; +Tb**; +Re**; +Hf**; +Te**; +Sr**;<br><br>--Sb**; --Cr**<br><br>-Ni**; -Cu**; -Sn**; -Ag**; -Au**

[a] Single "+" means positive $r_s < .20$; "++" means r_s from 0.20 to 0.29; "+++" means r_s from 0.30 to 0.39; "++++" means r_s from 0.40 to 0.49; (same ranges for "-" and "--", etc. negative r_s).

SI-2a Small datasets: Spearman rank correlations[a] between dust concentrations and heating fuel and heat distribution style (*indicates significant at $p < .05$; ** indicates significant at $p < .01$).

	Phthalates	BPAA (BPA analogues)	Aryl and Alkyl-Aryl Phosphates	Parabens and Triclosan	Musks
**Heating Fuel**					
**wood**	++DCHP*	0 compounds	++BtBPPP**<br>+BDPHP*<br>+tBPDPP*	0 compounds	0 compounds
**gas**	0 compounds	0 compounds	0 compounds	0 compounds	0 compounds
**electric**	0 compounds	+BPF*	0 compounds	0 compounds	+MK*<br>+MX*
**oil**	0 compounds	++BPTMC*	0 compounds	0 compounds	+MT*
**Heat Distribution Style**					
**forced air**	0 compounds	++++BPM**	0 compounds	0 compounds	0 compounds
**baseboard**	0 compounds	+BPB*	0 compounds	0 compounds	+MK*<br>+MX*
**fireplace**	0 compounds	+BPS*	++IDDPHP*	++MeP**<br>+PrP*	0 compounds
**stove**	++DMP**	0 compounds	++BDPHP**	0 compounds	0 compounds
**radiator**	+DCHP*	0 compounds	++BDPHP**<br>+24DIPPDPP & BIPPPP*	0 compounds	+MK**

[a] Single "+" means positive $r_s < .20$; "++" means r_s from 0.20 to 0.29; "+++" means r_s from 0.30 to 0.39; "++++" means r_s from 0.40 to 0.49.

SI-2b Small datasets: Spearman rank correlations[a] between dust concentrations and construction date, flooring and other characteristics.
(*indicates significant at $p < .05$; ** indicates significant at $p < .01$).

	**Phthalates (29 compounds in 126 homes)**	**BPA analogues (16 compounds in 115 homes)**	**Aryl and Alkyl-Aryl Phosphates (18 compounds in 139 homes)**	**Parabens and Triclosan (7 compounds in 259 homes)**	**Musks (13 compounds in 197 homes)**
**Construction date**	++DBzP+BzEHP**<br>--DBP**	+BPS*	--ODPP**<br>-TXP*<br>+IDDPHP*	+PrP **<br>+BuP*<br>+MeP*	---MX**<br>--MK**<br>-HHCB-lactone*<br>-ADBI*
**Municipal zone**	--DBP**<br>--BzBP*<br>-DIBP*<br>-DOP*	0 compounds	0 compounds	0 compounds	--ADBI**<br>--MK**<br>--ATII**<br>-MX*<br>-AHTN*
**Attached garage**	+DBzP+BzEHP*	0 compounds	0 compounds	0 compounds	0 compounds
**% Carpet**	0 compounds	++BPS**	0 compounds	+MeP**	+HHCB*
**% Hardwood**	++DIBP**	0 compounds	0 compounds	0 compounds	0 compounds
**% Vinyl**	0 compounds	0 compounds	++IDDPHP**	0 compounds	+HHCB*

[a] Single "+" means positive $r_s < .20$; "++" means r_s from 0.20 to 0.29; "+++" means r_s from 0.30 to 0.39; (same ranges for "-" and "--", etc. negative r_s).

SI-2c Small datasets: Spearman rank correlations[a] between dust concentrations and proximity to industry, inhabitants, and smoking behaviour (*indicates significant at $p < .05$; ** indicates significant at $p < .01$).

	**Phthalates**	**BPAA (BPA analogues)**	**Aryl and Alkyl-Aryl Phosphates**	**Parabens and Triclosan**	**Musks**
**# children**	0 compounds	0 compounds	0 compounds	0 compounds	0 compounds
**# dogs**	0 compounds	0 compounds	++tBPDPP*	0 compounds	0 compounds
**# cats**	0 compounds	0 compounds	0 compounds	0 compounds	+MT*
**# cigarette**	+DEP*	++BPAP**	+RBDP*	0 compounds	0 compounds
**# cigar**	0 compounds	++BPB*<br>++BPAP*	0 compounds	0 compounds	0 compounds
**Within 2 km of any industry**	++DMP*	0 compounds	0 compounds	0 compounds	0 compounds

[a] Single "+" means positive $r_s < .20$; "++" means r_s from 0.20 to 0.29.

SI-2d Small datasets: Spearman rank correlations[a] between concentrations and consumer products and dust loading rate (*indicates significant at $p < .05$; ** indicates significant at $p < .01$).

	**Phthalates**	**BPAA (BPA analogues)**	**Aryl and Alkyl-Aryl Phosphates**	**Parabens and Triclosan**	**Musks**
**Candles**	0 compounds	0 compounds	0 compounds	0 compounds	+ADBI**<br>+AHTN**<br>+HHCB*<br>+MK*<br>+HHCB-lactone*<br>+OTNE*
**Deodorizer**	0 compounds	0 compounds	+TIPPP*<br>+RBDP*<br>+IDDPHP*	0 compounds	+++OTNE**<br>++HHCB-lactone**<br>++HHCB**<br>++ATII**<br>+AHTN**
**Insecticides**	0 compounds	0 compounds	0 compounds	0 compounds	0 compounds
**Upholstery**	0 compounds	0 compounds	0 compounds	0 compounds	+OTNE**<br>+HHCB**
**Dust loading**	++DBP**<br><br>----DIDP**<br>--DUP **<br>--DNP**<br>--DINP**<br>--DDP**<br>--DBzP+BzEHP*<br>--B79P*<br>--DIHepP*<br>--DEHP*<br>--DnHepP*<br>-DOP*	--BPS**<br>--BPAF*	+ODPP*<br>-CDPP*	--BuP**<br>-MeP**	0 compounds

[a] Single "+" means positive $r_s < .20$; "++" means r_s from 0.20 to 0.29; "+++" means r_s from 0.30 to 0.39; (same ranges for "-" and "--", etc. negative r_s).

SI-3 Relationships between house characteristics (Spearman rho values and significance at $p < .05$* or $p < .01$**)

	Dust Loading Rate <80um	Candles	Cigarettes	Cigar/Pipe	Proximity to Industry	Municipal Zone	# Children	Deodorizers	Upholstery Treatments	Insecticide	# Dogs	# Cats	Attached Garage	Construction Date	% Vinyl floor	% Carpet	% Hardwood	Wood Heat	Gas Heat	Electric Heat	Oil Heat	Forced Air	Baseboard	Fireplace	Stove	Radiator
Dust Loading Rate <80 micron	1.000																									
Candles	.032	1.000																								
Cigarettes	.121**	-.052	1.000																							
Cigar/Pipe	.004	-.049	.121**	1.000																						
Proximity to Industry	.162**	-.024	.035	.027	1.000																					
Municipal Zone	.032	-.080*	.044	.034	.006	1.000																				
# Children	.021	-.032	.020	-.052	.030	.023	1.000																			
Deodorizers	.059	.402**	.025	-.009	.036	-.064*	-.058	1.000																		
Upholstery Treatments	.060	-.030	.035	.064*	.014	.067*	.068*	.092**	1.000																	
Insecticide	-.029	-.065*	.016	.026	.042	.115**	.028	-.086**	.195**	1.000																
# Dogs	.205**	.012	.074*	-.022	.079*	.054	.076*	.057	.039	.015	1.000															
# Cats	.137**	.034	.075*	.013	.070*	-.014	.050	.021	.023	-.038	-.026	1.000														
Attached Garage	-.106**	-.165**	-.012	-.013	-.059	.192**	.059	-.076*	.037	.061	.029	.014	1.000													
Construction Date	-.194**	-.004	-.027	-.023	-.055	.276**	.072*	-.001	.078*	-.043	.001	-.050	.437**	1.000												
% Vinyl floor	0.024	.023	.023	.010	.042	.037	.001	.149**	.034	.021	.031	.046	-.002	-.003	1.000											
% Carpet	.103**	-.034	.001	-.003	.001	.195**	-.068*	.031	.117**	.033	-.039	-.070*	.193**	.203**	-.091**	1.000										
% Hardwood	-.062*	-.018	-.024	-.021	-.015	-.198**	.042	-.034	-.120**	-.009	.060	.031	-.147**	-.248**	-.272**	-.793**	1.000									
Wood Heat	-.023	-.040	-.030	.010	-.036	.031	-.007	-.078*	-.010	.046	.017	.031	.023	-.072*	-.030	-.053	.062	1.000								
Gas Heat	.039	-.188**	.011	-.012	.046	.100**	.048	.107**	.163**	.044	.052	.013	.180**	.028	.088**	.218**	-.167**	-.239**	1.000							
Electric Heat	-.06	.195**	-.006	-.005	-.090**	-.188**	-.018	.082**	-.060	-.055	-.050	-.073*	-.120**	.025	-.012	-.228**	.179**	.009	-.480**	1.000						
Oil Heat	.012	.045	.017	.024	.010	.034	-.033	-.155**	-.102**	.001	-.054	.022	-.117**	-.125**	-.039	-.019	.017	.106**	-.510**	-.050	1.000					
Forced Air	.121**	-.118**	.041	.032	.036	.180**	.135**	-.172**	.103**	.154**	.051	.066*	.148**	.046	.028	.156**	-.126**	-.107**	.204**	-.190**	.051	1.000				
Baseboard	-.058	.143**	.004	.016	-.079*	-.166**	-.016	.036	-.031	-.048	-.050	-.081**	-.086**	.047	-.003	-.181**	.138**	.005	-.417**	.819**	-.080*	-.315**	1.000			
Fireplace	-.146**	-.043	-.047	.007	-.044	.157**	.031	-.024	.134**	.091**	.056	-.015	.268**	.232**	-.043	.086**	-.042	.424**	.098**	-.029	-.075*	.012	-.043	1.000		
Stove	.061	.032	-.005	-.027	-.010	.070*	-.024	-.075*	-.005	-.025	.012	.038	-.050	.020	-.048	-.057	.019	.270**	-.194**	.102**	.107**	-.037	.105**	-.059	1.000	
Radiator	.034	.074*	.017	-.016	-.020	-.079*	.053	.027	-.009	-.011	.024	.046	-.083**	-.147**	.006	-.033	.042	.062*	-.013	-.049	.150**	-.121**	-.029	-.034	.060	1.000

SI-4 Heating styles documented for CHDS homes, 2007-2010. Note that most homes use a combination of heating styles which is why the numbers do not add up to 100%.

	**Heating Fuel or Distribution Style Present**	
	**N**	**Percent**
Heating Fuel		
Wood	344	33.8%
Gas	740	72.7%
Electric	248	24.4%
Oil	94	9.2%
Heat Distribution Style		
Force Air	485	47.6%
Baseboard	181	17.8%
Fireplace	674	66.2%
Stove	55	5.4%
Radiator	20	2.0%

SI-5 Influence of carpets on dust loading rates (mg/m2/day) in Canadian House Dust Study (CHDS) homes located (top) more than 2 km from any industry; and (bottom) within 2 km radius of any industry. Table provides details for Figure 2 in main paper, showing median values for two size fractions (< 80 µm and 80-300 µm); N = number of homes in each quartile. "Fraction < 80 µm" refers to < 80 µm/< 300 µm, which positively correlates with % carpet in CHDS homes (Pearson r = +.40; *p* < .001; n=1022). The opposite trend is observed for 80-300 µm/< 300 µm (r = -.40; *p* < .001).

Proximity	% Carpet		< 80 µm ($mg/m^2/day$)	80-300 µm ($mg/m^2/day$)	Combined < 300 µm ($mg/m^2/day$)	Fraction < 80 µm
away from industry (> 2 km)	0-18	N	178	178		
		Median	3.8	4.8	8.6	44 wt%
	18-47	N	118	118		
		Median	4.8	4.2	9.0	53 wt%
	47-75	N	135	135		
		Median	5.1	3.9	9.1	56 wt%
	75-100	N	149	149		
		Median	5.8	3.4	9.2	63 wt%
close to industry (within < 2 km)	0-18	N	122	122		
		Median	5.9	6.2	12.1	49 wt%
	18-47	N	102	102		
		Median	9.5	6.7	16.2	59 wt%
	47-75	N	93	92		
		Median	7.8	5.2	13.0	60 wt%
	75-100	N	104	104		
		Median	7.6	4.8	12.4	61 wt%

SI-6 Concentrations and loadings (< 80 µm fraction) for BDE Flame retardants determined in subset of 642 Canadian House Dust Study samples (BDE = brominated diphenyl ethers). Units are µg/g for concentrations; µg/m2/day for loading rate, formatted in scientific notation. Note n = 305 homes for BDE-209 only. For CAS numbers and analytical details see Kubwabo, C., Fan, X., Rasmussen, P.E., X. Xia, X., Shang, H. (2015) Polybrominated Diphenyl Ethers and Novel Flame Retardants in the Canadian House Dust Study. Poster and Abstract presented at the Health Canada Science Forum, February 23-24, 2015, Ottawa (ON) Canada.

			Concentration			Loading Rate		
**Compound**	**% ≥LOD**	**LOD**	**50th %ile**	**95th %ile**	**Maximum**	**50th %ile**	**95th %ile**	**Maximum**
BDE-209	100	1.30E-02	1.13E+00	5.54E+00	4.74E+01	8.65E-03	8.40E-02	3.80E+00
BDE-100	100	7.11E-04	6.14E-02	3.59E-01	2.14E+00	4.09E-04	6.08E-03	8.14E-02
BDE-138	92	1.41E-03	7.52E-03	6.94E-02	2.60E+00	4.90E-05	1.11E-03	7.31E-03
BDE-153	100	6.64E-04	4.71E-02	3.10E-01	1.50E+00	3.45E-04	5.10E-03	6.18E-02
BDE-154	100	1.13E-03	2.83E-02	2.04E-01	1.03E+00	2.06E-04	3.28E-03	5.45E-02
BDE-17	95	4.00E-04	2.57E-03	1.87E-02	2.24E+00	1.70E-05	3.28E-04	4.07E-02
BDE-183	95	1.30E-03	1.76E-02	1.17E-01	9.33E-01	1.17E-04	1.70E-03	6.92E-02
BDE-28	99	6.00E-04	6.89E-03	4.13E-02	2.95E-01	4.40E-05	7.85E-04	6.87E-02
BDE-47	99	6.00E-04	2.24E-01	1.31E+00	7.40E+00	1.52E-03	2.39E-02	3.05E-01
BDE-66	95	5.89E-04	7.04E-03	1.65E-01	1.67E+00	4.50E-05	1.75E-03	2.61E-02
BDE-71	96	5.00E-04	6.96E-03	7.00E-02	7.67E-01	4.70E-05	1.01E-03	1.00E-02
BDE-85	99	8.79E-04	1.84E-02	1.36E-01	9.00E-01	1.21E-04	2.42E-03	2.32E-02
BDE-99	100	9.78E-04	2.49E-01	1.34E+00	6.18E+00	1.40E-03	2.69E-02	3.40E-01

SI-7 Concentrations and loadings (< 80 µm fraction) for non-BDE halogenated flame retardants determined in 642 Canadian House Dust Study samples. Units are µg/g for concentrations; µg/m^2/day for loading rate, formatted in scientific notation. For CAS numbers and analytical details see Fan, X.; Kubwabo, C., Rasmussen, P.E., Wu, F. (2016) Non-PBDE halogenated flame retardants in Canadian indoor house dust: sampling, analysis, and occurrence. Environmental Science and Pollution Research 23: 7998-8007.

			Concentration			Loading Rate		
**Compound**	**% ≥LOD**	**LOD**	**50th %ile**	**95th %ile**	**Maximum**	**50th %ile**	**95th %ile**	**Maximum**
(α+β)-TBECH	86	8.00E-04	4.03E-03	1.14E-01	1.95E+00	2.30E-05	2.08E-03	6.87E-02
(γ+δ)-TBECH	0	6.10E-04	<LOD	<LOD	<LOD	<LOD	<LOD	<LOD
(*syn+anti*)-DP	100	1.50E-03	1.32E-02	1.48E-01	2.51E+00	7.50E-05	1.94E-03	1.16E-01
α-TBECH	70	9.00E-04	2.14E-03	5.94E-02	1.18E+00	1.10E-05	1.10E-03	3.63E-02
anti-DP	100	1.90E-03	8.28E-03	1.01E-01	1.57E+00	4.10E-05	1.25E-03	5.08E-02
ATE	67	5.00E-04	9.40E-04	2.76E-02	1.06E+00	7.00E-06	2.52E-04	8.53E-02
β-TBECH	76	6.20E-04	1.81E-03	5.31E-02	7.77E-01	9.00E-06	9.27E-04	3.25E-02
BATE	39	4.80E-04	<LOD	4.64E-03	4.66E-02	<LOD	6.37E-05	3.09E-03
BTBPE	94	1.66E-03	9.75E-03	1.59E-01	2.39E+00	6.50E-05	2.58E-03	5.46E-02
DPTE	0	5.00E-04	<LOD	<LOD	<LOD	<LOD	<LOD	<LOD
EHTBB	97	1.46E-03	1.23E-01	1.83E+00	2.24E+01	8.98E-04	2.08E-02	4.41E-01
HBB	97	5.50E-04	1.08E-02	7.19E-02	4.31E-01	6.60E-05	1.34E-03	2.34E-02
PBB	58	8.10E-04	1.03E-03	7.72E-03	1.11E-01	3.00E-06	1.23E-04	3.72E-03
PBBA	0	1.64E-03	<LOD	<LOD	<LOD	<LOD	<LOD	<LOD
PBBB	0	1.00E-03	<LOD	<LOD	<LOD	<LOD	<LOD	<LOD
PBEB	14	5.80E-04	<LOD	1.64E-03	4.93E-02	<LOD	1.59E-05	6.10E-04
PBT	34	5.70E-04	<LOD	3.43E-03	1.09E-01	<LOD	3.89E-05	3.71E-03
syn-DP	100	1.20E-03	4.45E-03	4.87E-02	1.43E+00	2.00E-05	6.66E-04	6.54E-02
TBCT	79	9.40E-04	2.45E-03	2.09E-02	1.87E-01	1.40E-05	3.37E-04	3.12E-02
TBpX	11	5.60E-04	<LOD	1.09E-03	1.53E-01	<LOD	8.90E-06	3.36E-03

SI-8 Concentrations and loadings (< 80 µm fraction) for organophosphate esters determined in subset of 816 Canadian House Dust Study samples. Units are µg/g for concentrations; µg/m ^{2}/day for loading rate, formatted in scientific notation. For CAS numbers and analytical details see Fan, X., Kubwabo, C., Rasmussen, P.E., Wu, F. (2014) Simultaneous determination of thirteen organophosphate esters in settled indoor house dust and a comparison between two sampling techniques. Science of the Total Environment, 491-492: 80–86.

			**Concentration**			**Loading Rate**		
**Compound**	**% ≥LOD**	**LOD**	**50th %ile**	**95th %ile**	**Maximum**	**50th %ile**	**95th %ile**	**Maximum**
EHDPP	90	1.60E-01	4.90E-01	2.17E+00	1.05E+02	3.34E-03	4.42E-02	2.11E+00
TBOEP	100	4.30E-01	3.43E+01	1.04E+02	2.75E+02	2.24E-01	2.75E+00	2.61E+01
TCEP	97	7.00E-02	1.16E+00	9.42E+00	9.40E+01	8.47E-03	1.21E-01	2.78E+00
TCPP	95	1.10E-01	1.62E+00	1.87E+01	1.20E+02	1.14E-02	2.72E-01	8.56E+00
TCrP	100	3.00E-02	4.87E+00	1.78E+01	2.95E+02	2.99E-02	3.94E-01	3.64E+01
TDCPP	100	8.00E-02	3.08E+00	1.31E+01	1.39E+02	2.22E-02	2.27E-01	2.51E+01
TEP	15	9.00E-02	<LOD	3.30E-01	2.83E+00	<LOD	3.17E-03	3.86E-02
TiBP	13	4.00E-01	<LOD	9.66E-01	3.25E+00	<LOD	9.15E-03	1.38E-01
TMP	0	8.00E-02	<LOD	<LOD	<LOD	<LOD	<LOD	<LOD
TnBP	91	7.00E-02	3.10E-01	1.83E+00	8.56E+00	2.19E-03	2.50E-02	3.31E-01
TPeP	0	1.60E-01	<LOD	<LOD	<LOD	<LOD	<LOD	<LOD
TPHP	99	1.30E-01	1.75E+00	1.16E+01	9.06E+01	1.36E-02	1.81E-01	1.12E+01
TPrP	0	2.00E-01	<LOD	<LOD	<LOD	<LOD	<LOD	<LOD

SI-9 Concentrations and loadings (< 80 µm fraction) for pesticide compounds determined in Canadian House Dust Study samples from 913 homes. Units are µg/g for concentrations; µg/m^2/day for loading rate, formatted in scientific notation. For CAS numbers and analytical details see: Kubwabo, C., Rasmussen, P.E., Grenier, G. Occurrence of Selected Pesticides in Canadian House Dust. In: Organohalogen Compounds, 2017, 79: 175-178.

			Concentration			Loading rate		
**Compound**	**% ≥LOD**	**LOD**	**50th %ile**	**95th %ile**	**Maximum**	**50th %ile**	**95th %ile**	**Maximum**
Aldrin	5	0.005	<LOD	5.79E-03	2.84E-01	<LOD	8.00E-06	4.74E-03
Azinphos Methyl	31	0.0267	<LOD	7.38E+00	3.63E+02	<LOD	4.99E-02	2.92E+00
Chlorpyrifos	39	0.0016	<LOD	1.22E-01	2.84E+00	<LOD	1.00E-03	3.86E-02
cis-Chlordane	7	0.0025	<LOD	2.83E-02	8.64E+00	<LOD	1.05E-04	5.19E-01
cis-Nonachlor	5	0.0025	<LOD	<LOD	3.82E+00	<LOD	<LOD	8.67E-01
Diazinon	31	0.0016	<LOD	3.59E-01	6.05E+01	<LOD	3.07E-03	1.37E+01
Dieldrin	3	0.0125	<LOD	<LOD	1.01E+01	<LOD	<LOD	6.81E-02
HCB	6	0.0006	<LOD	9.16E-03	8.91E-01	<LOD	3.24E-05	2.15E-02
Heptachlor	6	0.0008	<LOD	7.78E-03	3.94E-01	<LOD	2.10E-05	7.42E-03
Heptachlor Epoxide	2	0.0003	<LOD	<LOD	4.88E-01	<LOD	<LOD	1.43E-03
Malathion	12	0.0032	<LOD	1.60E-02	1.01E+00	<LOD	1.38E-04	2.34E-02
Methyl Parathion	1	0.02	<LOD	<LOD	4.78E-02	<LOD	<LOD	4.04E-03
o,p'-DDD	6	0.005	<LOD	1.40E-02	2.54E+01	<LOD	8.25E-05	2.45E-01
o,p'-DDE	7	0.0004	<LOD	1.01E-02	3.89E+00	<LOD	5.63E-05	3.75E-02
o,p'-DDT	34	0.005	<LOD	6.10E-01	7.17E+01	<LOD	5.72E-03	4.52E-01
p,p'-DDD	46	0.005	<LOD	2.24E-01	1.45E+01	<LOD	3.45E-03	2.76E-01
p,p'-DDE	24	0.0013	<LOD	9.00E-02	3.42E+02	<LOD	1.61E-03	3.29E+00
p,p'-DDT	49	0.0063	<LOD	6.72E-01	4.07E+03	<LOD	7.85E-03	3.92E+01
Parathion	1	0.0533	<LOD	<LOD	1.48E-01	<LOD	<LOD	8.08E-04
Permethrin	67	0.005	1.64E-01	1.65E+01	3.24E+03	8.36E-04	1.49E-01	3.71E+01
Phorate	2	0.016	<LOD	<LOD	3.44E-01	<LOD	<LOD	1.87E-02
Terbufos	5	0.0064	<LOD	2.27E-03	3.85E-01	<LOD	1.13E-05	1.84E-02
trans-Nonachlor	7	0.0004	<LOD	2.68E-02	3.22E+02	<LOD	1.34E-04	1.96E+00
Triallate	2	0.0016	<LOD	<LOD	2.35E+00	<LOD	<LOD	2.08E-02

SI-10 Concentrations and loadings (< 80 µm fraction) for BPA, nonylphenols and octylphenol determined in 863 Canadian House Dust Study samples (n=813 for BPA). Units are µg/g for concentrations; µg/m^2/day for loading rate, formatted in scientific notation. For CAS numbers and analytical details see: Kubwabo, C., Rasmussen, P.E., Fan, X., Kosarac, I., Grenier G., Coleman, K. (2016a) Simultaneous quantification of bisphenol A, alkylphenols and alkylphenol ethoxylates in indoor dust by gas chromatography-tandem mass spectrometry and a comparison between two sampling techniques. Analytical Methods 8: 4093-4100.

			Concentration			Loading Rate		
**Compound**	**% ≥LOD**	**LOD**	**50th %ile**	**95th %ile**	**Maximum**	**50th %ile**	**95th %ile**	**Maximum**
BPA	100	1.46E-01	3.53E+00	2.78E+01	1.07E+02	1.87E-02	3.50E-01	3.07E+00
Branched-NP	100	1.00E-01	1.03E+01	7.32E+01	8.84E+02	5.84E-02	1.10E+00	1.51E+01
n-NP	96	1.00E-02	7.60E-01	5.09E+00	4.87E+01	3.66E-03	9.54E-02	6.70E-01
NP_1EO	100	1.00E-01	3.67E+00	3.05E+01	1.38E+02	2.20E-02	5.37E-01	5.13E+00
NP_2EO	96	1.60E-01	6.07E+00	5.31E+01	2.04E+02	3.35E-02	8.65E-01	5.96E+00
4-*tert*-OP	99	3.00E-02	3.00E-01	1.13E+01	2.51E+02	2.34E-03	2.45E-01	5.13E+00

SI-11 Concentrations and loadings (< 80 µm fraction) for phthalates determined in 128 Canadian House Dust Study samples. Units are µg/g for concentrations; µg/m2/day for loading rate, formatted in scientific notation. For CAS numbers and analytical details see: Kubwabo, C., Rasmussen, P.E., Fan, X., Kosarac, I., Wu, F., Zidek, A., Kuchta, S.L. (2013) Analysis of selected phthalates in Canadian indoor dust collected using household vacuum and standardized sampling techniques. Indoor Air, 23 (6), pp. 506-514; and Kubwabo, C., Fan, X., Pat E. Rasmussen, P.E., Wu, F., Kosarac, I. (2016b) Expanding the Number of Phthalates Monitored in House Dust. International Journal of Environmental Analytical Chemistry 96: 667-681.

			Concentration			Loading Rate		
Compound	% ≥LOD	LOD	50th %ile	95th %ile	Maximum	50th %ile	95th %ile	Maximum
B79P	96	0.12	1.07E+00	7.88E+00	2.90E+01	1.27E-02	1.10E-01	5.82E-01
BCHxP	0	0.18	<LOD	<LOD	<LOD	<LOD	<LOD	<LOD
BzBP	100	0.14	4.82E+01	5.31E+02	9.44E+02	4.92E-01	1.46E+01	5.61E+01
DAP	0	0.11	<LOD	<LOD	<LOD	<LOD	<LOD	<LOD
DBP	99	0.3	2.24E+01	1.19E+02	4.82E+02	1.96E-01	5.87E+00	2.34E+01
DBzP+BzEHP	84	0.36	3.14E+00	1.74E+01	6.12E+01	2.49E-02	3.84E-01	2.79E+00
DCHP	59	0.17	1.90E-01	1.87E+00	1.34E+01	5.90E-04	2.62E-02	2.19E-01
DDP	98	0.18	1.73E+00	1.09E+02	8.48E+02	1.96E-02	3.64E-01	4.00E+00
DEHP	100	2.93	4.06E+02	2.03E+03	3.84E+03	3.43E+00	4.80E+01	1.35E+02
DEP	95	0.47	1.80E+00	1.70E+01	1.93E+02	1.41E-02	4.51E-01	1.88E+00
DHP	92	0.11	4.90E-01	1.02E+01	4.68E+01	5.40E-03	1.46E-01	3.73E+00
DIBP	99	0.51	6.12E+00	3.07E+01	6.93E+01	5.95E-02	1.14E+00	5.73E+00
DIDP	100	1.22	7.09E+01	4.24E+02	1.43E+03	7.87E-01	6.73E+00	1.40E+01
DIHepP	99	0.52	1.69E+01	2.37E+02	6.44E+02	2.04E-01	3.03E+00	1.04E+01
DiHxP	90	0.42	1.97E+00	1.05E+02	5.21E+02	2.30E-02	1.18E+00	4.15E+01
DINP	100	1.07	1.13E+02	5.22E+02	1.36E+03	1.27E+00	1.37E+01	2.85E+01
DIOP	90	1.09	6.26E+00	3.26E+01	1.17E+03	5.10E-02	1.26E+00	1.09E+01
DiTDP	23	1.14	<LOD	4.34E+00	3.29E+01	<LOD	6.36E-02	1.63E-01
DMCHP1	30	0.04	<LOD	2.76E-01	4.06E+00	<LOD	7.98E-03	5.78E-02
DMCHP2	88	0.06	4.90E-01	9.51E+00	2.84E+01	5.09E-03	1.02E-01	3.95E-01
DMP	92	0.04	1.30E-01	1.72E+00	2.24E+01	1.39E-03	3.20E-02	2.92E-01
DMT	0	0.13	<LOD	<LOD	<LOD	<LOD	<LOD	<LOD
DnHepP	99	0.16	3.06E+00	5.48E+01	2.64E+02	4.10E-02	6.88E-01	2.50E+00
DNP	99	0.16	4.93E+00	1.08E+02	4.30E+02	5.45E-02	9.05E-01	2.88E+00
DOP	98	0.12	1.12E+00	5.32E+01	3.92E+02	1.20E-02	1.75E-01	1.85E+00
DPrP	0	0.12	<LOD	<LOD	<LOD	<LOD	<LOD	<LOD
DTDP	0	0.18	<LOD	<LOD	<LOD	<LOD	<LOD	<LOD
DUP	100	0.15	3.41E+00	5.10E+01	1.74E+02	4.50E-02	5.54E-01	8.59E-01
IBCHxP	0	0.08	<LOD	<LOD	<LOD	<LOD	<LOD	<LOD

SI-12 Concentrations and loadings (< 80 µm fraction) for synthetic musks determined in 198 Canadian House Dust Study samples. Units are µg/g for concentrations; µg/m^2/day for loading rate, formatted in scientific notation. For CAS numbers and analytical details see: Kubwabo, C., Fan, X., Rasmussen, P.E., Wu, F. (2012) Determination of synthetic musk compounds in indoor house dust by gas chromatography-ion trap mass spectrometry. Analytical and Bioanalytical Chemistry 404 (2): 467-477.

			Concentration			Loading Rate		
**Compound**	**% ≥LOD**	**LOD**	**50th %ile**	**95th %ile**	**Maximum**	**50th %ile**	**95th %ile**	**Maximum**
ADBI	27	2.00E-02	<LOD	1.10E-01	2.55E+00	<LOD	1.74E-03	1.43E-02
AHMI	21	2.20E-02	<LOD	8.85E-02	1.04E+01	<LOD	1.41E-03	3.62E-02
AHTN	100	2.70E-02	5.11E-01	2.95E+00	2.37E+01	5.56E-03	6.42E-02	2.38E-01
ATII	40	1.30E-02	<LOD	9.82E-02	4.11E-01	<LOD	2.62E-03	4.38E-02
DPMI	0	3.90E-02	<LOD	<LOD	<LOD	<LOD	<LOD	<LOD
HHCB	100	2.90E-02	9.49E-01	8.11E+00	3.45E+01	1.14E-02	1.78E-01	1.08E+00
HHCB-lactone	100	4.80E-02	4.59E-01	2.49E+00	8.49E+00	5.14E-03	6.42E-02	1.79E-01
MA	0	1.80E-02	<LOD	<LOD	<LOD	<LOD	<LOD	<LOD
MK	79	1.30E-02	4.93E-02	2.56E-01	2.22E+00	3.95E-04	7.43E-03	1.20E-01
MM	0	3.80E-02	<LOD	<LOD	<LOD	<LOD	<LOD	<LOD
MT	27	2.10E-02	<LOD	2.92E-01	1.56E+00	<LOD	4.21E-03	2.46E-02
MX	95	1.20E-02	4.59E-02	3.45E-01	6.36E+00	5.10E-04	1.54E-02	8.22E-02
OTNE	91	3.50E-02	2.68E-01	2.67E+00	2.11E+01	2.97E-03	7.44E-02	2.20E-01

SI-13 Concentrations and loadings (< 80 µm fraction) for parabens and triclosan determined in 263 Canadian House Dust Study samples. Units are µg/g for concentrations; µg/m^2/day for loading rate, formatted in scientific notation. For CAS numbers and analytical details see: Fan, X., Kubwabo, C., Rasmussen, P., Jones-Otazo, H. (2010) Simultaneous quantitation of parabens, triclosan, and methyl triclosan in indoor house dust using solid phase extraction and gas chromatography-mass spectrometry. Journal of Environmental Monitoring 12 (10): 1891-1897.

			Concentration			Loading Rate		
**Compound**	**% ≥LOD**	**LOD**	**50th %ile**	**95th %ile**	**Maximum**	**50th %ile**	**95th %ile**	**Maximum**
BuP	74	1.00E-02	8.62E-02	5.12E-01	1.21E+01	3.47E-04	7.36E-03	2.66E-01
BzP	0	8.00E-03	<LOD	<LOD	<LOD	<LOD	<LOD	<LOD
EtP	84	6.50E-03	4.62E-02	3.41E-01	1.53E+00	3.02E-04	6.08E-03	3.62E-02
MeP	100	7.00E-03	1.48E+00	5.01E+00	1.06E+01	9.89E-03	1.02E-01	5.24E-01
MTCS	0	9.20E-03	<LOD	<LOD	<LOD	<LOD	<LOD	<LOD
PrP	100	8.70E-03	6.81E-01	5.03E+00	1.29E+01	4.21E-03	7.38E-02	3.34E-01
Triclosan	100	8.70E-03	4.57E-01	2.55E+00	7.85E+00	3.26E-03	4.32E-02	2.45E-01

SI-14 Concentrations and loadings (< 80 µm fraction) for BPA analogues (BPAA) determined in 119 Canadian House Dust Study samples. Units are µg/g for concentrations; µg/m^2/day for loading rate, formatted in scientific notation. For CAS numbers and analytical details see: Fan, X., Katuri, G.P. Caza, A., Rasmussen, P.E., Kubwabo, C. (2021) Simultaneous measurement of 16 bisphenol analogues in house dust and evaluation of two sampling techniques. Emerging Contaminants, 2021, 7:1-9.

			Concentration			Loading Rate		
**Compound**	**% ≥LOD**	**LOD**	**50th %ile**	**95th %ile**	**Maximum**	**50th %ile**	**95th %ile**	**Maximum**
BPAF	15	3.00E-03	<LOD	8.00E-03	1.90E-02	<LOD	4.16E-05	3.51E-04
BPAP	21	3.00E-03	<LOD	8.00E-03	2.20E-02	<LOD	1.46E-04	1.82E-03
BPB	21	2.00E-03	<LOD	5.00E-03	8.10E-02	<LOD	9.04E-05	5.04E-03
BPBP	0	8.00E-03	<LOD	<LOD	<LOD	<LOD	<LOD	<LOD
BPC	0	3.00E-03	<LOD	<LOD	<LOD	<LOD	<LOD	<LOD
BPC2	1	3.00E-03	<LOD	<LOD	3.00E-03	<LOD	<LOD	6.00E-06
BPE	0	1.00E-03	<LOD	<LOD	<LOD	<LOD	<LOD	<LOD
BPF	85	7.00E-03	1.23E-01	8.20E-01	2.69E+00	8.28E-04	1.52E-02	7.90E-02
BPG	0	6.00E-03	<LOD	<LOD	<LOD	<LOD	<LOD	<LOD
BPM	77	5.00E-03	2.80E-02	2.21E-01	9.08E-01	1.91E-04	5.53E-03	4.34E-02
BPP	0	7.00E-03	<LOD	<LOD	<LOD	<LOD	<LOD	<LOD
BPPH	0	7.00E-03	<LOD	<LOD	<LOD	<LOD	<LOD	<LOD
BPS	85	1.70E-02	2.42E-01	2.28E+00	3.51E+01	1.56E-03	6.33E-02	5.57E-01
BPTMC	49	5.00E-03	<LOD	6.30E-02	3.09E-01	<LOD	2.36E-03	1.84E-02
BPZ	0	6.00E-03	<LOD	<LOD	<LOD	<LOD	<LOD	<LOD

SI-15 Concentrations and loadings (< 80 µm fraction) for aryl and alkyl-aryl phosphates determined in 143 Canadian House Dust Study samples. Units are µg/g for concentrations; µg/m^2/day for loading rate, formatted in scientific notation. For CAS numbers and analytical details see: Kubwabo C, Fan X, Katuri GP, Habibagahi A, Rasmussen PE. Occurrence of aryl and alkyl-aryl phosphates in Canadian house dust. Emerging Contaminants. 2021 Jan 1;7:149-59.

			Concentration			Loading Rate		
**COMPOUND**	**% ≥LOD**	**LOD**	**50th %ile**	**95th %ile**	**Maximum**	**50th %ile**	**95th %ile**	**Maximum**
24DIPPDPP & BIPPPP	100	1.97E-02	3.75E-01	5.71E+00	4.60E+01	3.46E-03	5.72E-02	3.67E+00
B24DIPPPP	0	1.55E-02	<LOD	<LOD	<LOD	<LOD	<LOD	<LOD
BDPHP	31	5.60E-03	<LOD	1.85E-02	8.78E-02	<LOD	6.59E-04	7.03E-03
BPA-DP	69	1.33E-02	3.01E-02	3.15E-01	8.18E-01	1.74E-04	6.48E-03	6.55E-02
BtBPPP	51	2.56E-02	2.58E-02	1.41E-01	4.20E-01	3.10E-05	4.24E-03	2.02E-02
CDPP	99	1.22E-02	9.07E-02	9.84E-01	5.77E+01	9.29E-04	1.97E-02	2.38E+00
DBPHP	0	4.00E-03	<LOD	<LOD	<LOD	<LOD	<LOD	<LOD
DDDPP	0	1.51E-02	<LOD	<LOD	<LOD	<LOD	<LOD	<LOD
DPTDP	0	1.12E-02	<LOD	<LOD	<LOD	<LOD	<LOD	<LOD
IDDPHP	100	2.23E-02	1.24E+00	7.85E+00	1.79E+01	1.26E-02	2.07E-01	7.02E-01
IPDPP	0	5.50E-03	<LOD	<LOD	<LOD	<LOD	<LOD	<LOD
IPPDPP	100	1.92E-02	3.24E-01	7.00E+00	5.29E+01	2.94E-03	7.66E-02	4.22E+00
ODPP	60	1.62E-02	2.13E-02	1.86E-01	6.54E-01	9.60E-05	4.97E-03	8.67E-02
RBDP	78	4.90E-03	1.45E-02	1.11E-01	3.07E-01	1.00E-04	3.27E-03	6.96E-03
T4tBPP	0	1.39E-02	<LOD	<LOD	<LOD	<LOD	<LOD	<LOD
tBPDPP	92	1.53E-02	5.06E-02	3.46E-01	3.33E+00	4.42E-04	1.01E-02	4.52E-02
TIPPP	76	4.24E-02	7.18E-02	6.26E-01	5.27E+01	4.01E-04	1.08E-02	4.20E+00
TXP	85	8.00E-03	2.24E-02	2.63E-01	1.01E+01	1.89E-04	3.07E-03	9.76E-02

SI-16 Total concentrations* (< 80 µm fraction) for metals and metalloids determined in Canadian House Dust Study samples (n=1025 homes). See main text for references.

			Percentiles								
**Element**	**N**	**Units**	**10**	**25**	**50**	**75**	**90**	**95**	**97.5**	**98**	**99**
**Ag**	1025	µg/g	0.69	1.09	1.84	3.21	5.94	9.33	12.7	14.2	34.0
**Al**	1025	%	0.91	1.15	1.51	1.95	2.50	2.90	3.2	3.32	3.60
**As**	1025	µg/g	3.02	5.20	9.00	15.2	26.5	40.6	54.0	57.5	71.6
**Au**	961	ng/g	191	301	516	987	1840	2839	4159	4527	7595
**B***	1025	µg/g	28.1	41.8	65.3	126	228	329	430	470	592
**Ba**	1025	µg/g	165	216	277	361	452	528	685	774	958
**Be**	1025	µg/g	0.20	0.30	0.40	0.55	0.70	0.85	1.00	1.07	1.47
**Bi**	1025	µg/g	0.80	1.20	2.42	5.61	13.2	21.4	38.0	40.5	64.6
**Br**	963	µg/g	18.9	22.6	29.4	43.2	93.0	148	302	347	558
**C**	1011	%	21.6	26.0	29.3	33.0	35.8	37.0	38.4	39.0	40.4
**Ca**	1025	%	2.42	3.49	4.83	6.10	7.82	9.02	10.0	10.3	11.1
**Cd**	1025	µg/g	1.50	2.20	3.50	5.90	11.1	17.2	25.6	29.1	51.5
**Co**	1025	µg/g	3.30	4.20	5.60	7.80	13.6	19.1	24.8	27.6	35.2
**Cr**	1025	µg/g	58.0	74.6	99.0	136	177	214	268	292	432
**Cs**	1025	µg/g	0.33	0.42	0.53	0.68	0.93	1.14	1.35	1.44	1.87
**Cu**	1025	µg/g	115	147	199	291	449	657	857	1025	1811
**Fe**	1025	%	0.62	0.82	1.06	1.42	1.87	2.27	2.71	2.91	3.62
**Ga**	1025	µg/g	1.50	2.30	3.25	4.43	6.00	7.20	8.60	9.32	10.7
**Ge**	1025	µg/g	<LOD	<LOD	<LOD	0.20	0.59	0.80	0.90	1.00	1.10
**Hf**	1025	µg/g	1.53	3.00	5.00	8.59	13.0	18.0	23.0	25.0	31.7
**Hg**	995	µg/g	0.21	0.37	0.68	1.30	2.40	3.74	5.38	5.94	8.46
**K**	1025	%	0.54	0.66	0.79	0.94	1.11	1.22	1.35	1.40	1.57
**Li**	1025	µg/g	4.00	5.10	6.50	8.40	10.9	12.9	15.0	15.6	20.5
**Mg**	1025	%	0.45	0.62	0.87	1.26	1.79	2.18	2.56	2.81	3.14
**Mn**	1024	µg/g	149	197	267	363	495	597	669	705	1103
**Mo**	1025	µg/g	1.00	2.00	2.70	4.00	5.40	8.00	13.2	14.7	20.6
**Na**	1021	%	1.09	1.34	1.70	2.32	3.22	4.22	5.40	6.27	8.18
**Nb**	1025	µg/g	0.40	1.10	2.10	3.38	5.07	6.47	7.94	8.20	9.65
**Ni**	1025	µg/g	36.9	47.0	62.4	94.5	198	322	501	613	856
**P**	818	%	0.06	0.08	0.106	0.13	0.16	0.195	0.24	0.25	0.35
**Pb**	1025	µg/g	48.6	66.3	100	173	357	760	1330	1554	2027
**Rb**	1025	µg/g	10.8	14.2	18.7	24.2	30.9	35.1	41.7	43.8	51
**Re**	1025	µg/g	<LOD	0.002	0.003	0.004	0.01	0.02	0.03	0.03	0.05
**S**	818	%	0.52	0.63	0.78	1.03	1.39	1.78	2.23	2.40	2.85
**Sb**	1025	µg/g	4.70	6.30	8.50	12.3	20.8	32.0	51.6	63.7	166
**Se**	1025	µg/g	<LOD	0.50	0.85	1.40	2.50	3.4	4.40	4.75	6.90
**Sn**	1025	µg/g	9.00	13.0	20.0	32.5	53.0	75.7	108	120	193
**Sr**	1025	µg/g	84.6	109	142	193	244	310	365	374	455
**Ta**	1025	µg/g	<LOD	<LOD	<LOD	<LOD	0.37	0.60	0.82	0.90	1.29
**Te**	1025	µg/g	0.46	0.36	0.18	0.60	1.00	1.30	1.54	1.65	2.07
**Ti**	818	%	0.14	0.17	0.21	0.27	0.34	0.40	0.48	0.52	0.62
**Tl**	1025	µg/g	0.06	0.08	0.10	0.14	0.17	0.20	0.23	0.25	0.30
**V**	1025	µg/g	11.0	14.0	18.0	25.0	34.0	45.0	55	58.0	72.2
**Zn**	1025	µg/g	456	559	726	964	303	1630	2041	2157	2637

*Boron (B) is the only element for which results are semi-quantitative (0.07 M HCl was used to determine bioaccessible boron)

SI-17. Chemicals and building materials lacking associations.

Certain chemicals lacked any correlation with house characteristics in the CHDS, likely because the study questionnaire did not capture the indoor uses or sources of the chemical. For example, triclosan displayed 100% detection frequency in CHDS samples [Fan et al., 2010] but displayed no correlations with house characteristics (Table 2). Another example is Zn which was enriched in indoor dust in the CHDS and many other studies [Beauchemin et al., 2014; Rasmussen et al., 2004, 2013, 2021], but displays very few correlations with house characteristics (two occurrences in Table 4 only). This lack of correlation was likely due to the numerous applications of Zn in residential environments including pigments, resins, extenders, drying agents for paints and coatings, plasticisers, adhesives, pharmaceuticals, pesticides, cosmetics, fire retardants, furniture and cookware [Blount, 1988; Graedel et al., 2013; Beauchemin et al., 2014]. Likewise, a wide range of other metal(loid)s are used in adhesives, extenders, fillers, resins and flame retardant coatings [Blount, 1988; Cahill et al., 1989; CPSC, 2016], and these uses would be difficult to capture using survey questionnaires.

Vinyl flooring does not appear in Table 4 or Table 5 because %vinyl does not correlate with either construction date (p =.91) or municipal zone (p =.25). Two OPEs correlated with %vinyl (TBOEP and TnBP at $p < .05$; SI-1b); the emerging aryl phosphate compound IDDPHP correlates with %vinyl ($p < .01$; SI-2b); and BPA correlates positively with %vinyl (SI-1b). As mentioned in the main text (Section 3.1.1), there were other house characteristics that correlated with %vinyl (deodorizers and gas heating; SI-3). The potential overlapping influence of these other house characteristics need to be considered when assessing the influence of vinyl flooring on dust chemistry.

References

S. Beauchemin, P.E. Rasmussen, T. MacKinnon, M. Chenier, K. Boros, Zinc in house dust: Speciation, bioaccessibility, and impact of humidity, Environmental science and technology. 48 (16) (2014) 9022-9029. https://doi.org/10.1021/es5018587.

D.H. Blount, Process for the production of flame-retardant polyurethane products. United States Patent number: 4778844. https://patents.justia.com/patent/4778844, 1988, (accessed 20 December 2021).

D.G. Cahill, H.E. Fischer, S.K. Watson, R.O. Pohl, G.A. Slack, 1989. Thermal properties of boron and borides. Physical Review B. 40 (5). https://doi.org/10.1103/PhysRevB.40.3254.

CPSC (Consumer Product Safety Commission), CPSC Staff Statement on the Toxicology Excellence for Risk Assessment Report, "Final Report for CPSC Task 14". https://www.cpsc.gov/s3fs-public/ManufacturedWoodsTERATask14Report.pdf, 2016, (accessed 20 December 2021).

X. Fan, C. Kubwabo, P.E. Rasmussen, H. Jones-Otazo, Simultaneous quantitation of parabens, triclosan, and methyl triclosan in indoor house dust using solid phase extraction and gas chromatography-mass spectrometry, Journal of Environmental Monitoring. 12 (10) (2010) 1891-1897. https://doi.org/10.1039/c0em00189a.

T.E. Graedel, E.M. Harper, N.T. Nassar, B.K. Reck, On the materials basis of modern society, Proceedings of the National Academy of Sciences. 112 (20) (2013) 6295-6300. https://doi.org/10.1073/pnas.1312752110.

P.E. Rasmussen, C. Levesque, O. Butler, M. Chénier, H.D. Gardner, Selection of metric for indoor-outdoor source apportionment of metals in $PM_{2.5}$: mg/kg versus ng/m^3, Indoor air, 32 (1) (2022) 12924. https://doi.org/10.1111/ina.12924.

P.E. Rasmussen, C. Levesque, M. Chénier, H.D. Gardner, H. Jones-Otazo, S. Petrovic, Canadian House Dust Study: Population-based concentrations, loads and loading rates of arsenic, cadmium, chromium, copper, nickel, lead, and zinc inside urban homes, Science of the Total Environment, 443 (2013) 520–529. https://doi.org/10.1016/j.scitotenv.2012.11.003.

P.E. Rasmussen PE, Elements and Their Compounds in Indoor Environments, in: E. Merian, M. Anke, M. Ihnat, M. Stoeppler (Eds), Elements and Their Compounds in the Environment: Occurrence, Analysis and Biological Relevance, Second Edition, WILEY-VCH Verlag GmbH & Co. KGaA, 2004, pp. 215-234. https://doi.org/10.1002/9783527619634.ch11.
